# DOT1L inhibition exerts the anti-tumor effect by activating interferon signaling in breast cancer cells

**DOI:** 10.1186/s13148-025-02017-5

**Published:** 2025-11-26

**Authors:** Ayano Yoshido, Kazuya Ishiguro, Hiroshi Kitajima, Takeshi Niinuma, Kohei Kumegawa, Masaki Maezawa, Tomohide Tsukahara, Mutsumi Toyota, Akira Yorozu, Hajime Sasaki, Eiichiro Yamamoto, Masahiro Kai, Masashi Idogawa, Toshihiko Torigoe, Hiroshi Nakase, Reo Maruyama, Hiromu Suzuki

**Affiliations:** 1 Division of Molecular Biology, Department of Biochemistry, Sapporo Medical University School of Medicine, S1, W17, Chuo-ku, Sapporo, 060-8556 Japan; 2 Cancer Cell Diversity Project, Next-Ganken program, Japanese Foundation for Cancer Research, Tokyo, Japan; 3 Division of Cancer Epigenomics, Cancer Institute, Japanese Foundation for Cancer Research, Tokyo, Japan; 4 Laboratory of OSG Veterinary Science for Global Disease Management, Graduate School of Agricultural and Life Sciences, The University of Tokyo, Tokyo, Japan; 5 Department of Pathology, Sapporo Medical University School of Medicine, Sapporo, Japan; 6 Division of Gastroenterology and Hepatology, Department of Internal Medicine, Sapporo Medical University School of Medicine, Sapporo, Japan; 7 Division of Medical Genome Sciences, Department of Genomic and Preventive Medicine, Sapporo Medical University School of Medicine, Sapporo, Japan

**Keywords:** DOT1L, Interferon, Immune response, DNA damage, STING

## Abstract

**Background:**

DOT1L, a histone H3 lysine 79 (H3K79) methyltransferase, is a potential therapeutic target in various malignancies. In the present study, we aimed to clarify the anti-tumor effect of DOT1L inhibition in breast cancer.

**Methods:**

Estrogen receptor (ER)-positive/HER2-negative breast cancer cells (MCF7) and ER-negative/HER2-positive cells (SKBR3) were treated with a DOT1L inhibitor (SGC0946, EPZ-5676), after which colony formation assays, cell cycle assays, flow cytometry, gene expression microarray analysis, chromatin immunoprecipitation sequencing (ChIP-seq) and single-cell Assay for Transposase-Accessible Chromatin sequencing (scATAC-seq) were performed. Genetic ablation of STING was performed using the CRISPR/Cas9 system.

**Results:**

Treatment with a DOT1L inhibitor suppressed proliferation and induced cell cycle arrest and apoptosis in both ER-positive/HER2-negative and ER-negative/HER2-positive cells. Transcriptome and epigenome analysis revealed that DOT1L inhibition activated transcription of a number of interferon (IFN)-related genes (IRGs) in breast cancer cells. We also found that DOT1L inhibition upregulated type I and type III IFNs as well as cell surface human leukocyte antigen (HLA) class I expression. Notably, DOT1L inhibition induced DNA damage and upregulated levels of cytoplasmic DNA in breast cancer cells. CRISPR/Cas9-mediated knockout of STING in breast cancer cells significantly suppressed the IFN signaling activated by DOT1L inhibition and attenuated the anti-tumor effect. Moreover, scATAC-seq analysis revealed that DOT1L inhibition suppressed expression of ERBB2 in HER2-positive breast cancer cells.

**Conclusion:**

These findings suggest that the anti-breast cancer effect of DOT1L inhibition is mediated by multiple mechanisms, including activation of innate immune signaling.

**Supplementary Information:**

The online version contains supplementary material available at 10.1186/s13148-025-02017-5.

## Introduction

Breast cancer is one of the most frequently occurring and lethal diseases in women. Indeed, there were 2.30 million new cases and 665,000 breast cancer-related deaths among women in 2022 [1]. At present, breast cancer is treated with surgery, chemotherapy, radiotherapy, endocrine therapy and molecular-targeted therapy. Clinically, the breast cancer subtypes are determined based on human epidermal receptor 2 (HER2) and hormone receptor expression, and the subtypes are associated with patient prognosis [2]. Early-stage breast cancer is treated with surgery; however, post-operative recurrence or cosmetic problems often remain [3]. Alternative drug treatment, including use of immune checkpoint inhibitors, improves patient prognosis, but drug resistance arises and impedes treatment [4]. Consequently, a novel therapeutic target for breast cancer is desired.

Epigenetic alterations, including aberrant histone modifications, play major roles in the initiation and progression of various human malignancies [5, 6]. Dot1-like protein (DOT1L) is a methyltransferase that catalyzes mono-, di- and trimethylation at histone H3 lysine 79 [7]. In mammals, DOT1L plays an essential role in normal development, hematopoiesis, cardiac function and leukemogenesis [8–11]. In mixed lineage leukemia (MLL)-rearranged leukemia, MLL fusion proteins interact with DOT1L and transcriptionally activate target oncogenes that drive leukemia development [12]. DOT1L inhibitors thus exert selective and strong anti-tumor effects against MLL-rearranged leukemia [13, 14]. DOT1L is also considered to be a potential therapeutic target in other malignancies. For instance, we previously showed that DOT1L inhibition suppresses proliferation of multiple myeloma cells by downregulating MYC-IRF4 signaling [15]. Increased expression of DOT1L is a prognostic factor in ovarian cancer, where DOT1L promotes cancer cell proliferation and drug resistance [16, 17]. Moreover, DOT1L promotes neuroblastoma by acting as a co-factor in N-Myc-mediated transcriptional activation of its target genes, and it promotes cell cycling by transcriptionally activating c-Myc in colorectal cancer cells [18, 19]. DOT1L also cooperates with androgen receptor (AR) to upregulate Myc in AR-positive prostate cancer [20].

Recent studies suggest DOT1L is an exploitable therapeutic target in breast cancer. Expression of DOT1L is higher in breast cancers than in normal breast tissues, and inhibition of DOT1L suppresses proliferation of breast cancer cells [21]. Through cooperation with c-Myc and p300 acetyltransferase, DOT1L promotes epithelial-mesenchymal transition (EMT) in breast cancer cells [22]. In addition, a recent study revealed that DOT1L is a novel cofactor of estrogen receptor α (ERα), acting to regulate estrogen target genes, and that DOT1L inhibition suppresses proliferation of antiestrogen-resistant breast cancer cells [23]. DOT1L was also shown to be a cancer stem cell (CSC) regulator in triple-negative breast cancer (TNBC), and its inhibition suppressed in vivo tumor growth and metastasis by decreasing TNBC CSCs [24].

In the present study, we identified a novel mechanism by which DOT1L inhibition exerts its anti-breast cancer effects. We show that DOT1L inhibition promotes DNA damage and activates an interferon (IFN) response in ER-positive breast cancer cells as well as in HER2-positive cancer cells. Genetic ablation of STING significantly suppressed the IFN signaling, suggesting that DOT1L inhibition triggers IFN signaling via the STING pathway. We also demonstrate that DOT1L inhibition suppresses proliferation of HER2-positive breast cancer cells, at least in part, by suppressing ERBB2 expression.

## Materials and methods

### Cell lines and reagents

Breast cancer cell lines (MCF7 and SKBR3) were obtained and cultured as described previously [25]. They were checked for mycoplasma contamination using an EZ-PCR Mycoplasma Detection Kit (Biological Industries, Beit HaEmek, Israel) and were confirmed to be negative. A DOT1L inhibitor, SGC0946, was purchased from Sigma-Aldrich (St. Louis, MO, USA). Another DOT1L inhibitor, EPZ-5676, was purchased from Chemietek (Indianapolis, IN, USA). Cells were treated with SGC0946 (1 µM), EPZ-5676 (1 µM) or DMSO for up to 15 days, replacing the medium and drug every 3 days.

### Western blot analysis

For western blot analysis, cells were treated with the aforementioned drugs for 6–12 days as described above. Total proteins were then extracted using Cell Lysis Buffer (#9803, Cell Signaling Technology, Danvers, MA, USA). Cytoplasmic and nuclear proteins were extracted using an SF PTS Kit (GL Sciences, Tokyo, Japan) as described previously [26]. Rabbit anti-Stat1 mAb (1:1000 dilution, #14994; Cell Signaling Technology), rabbit anti-phospho-Stat1 mAb (1:1000 dilution, #9167; Cell Signaling Technology), rabbit anti-histone H3 mAb (1:2000 dilution, #4499; Cell Signaling Technology), rabbit anti-histone γH2AX mAb (1:1000 dilution, #9718; Cell Signaling Technology), rabbit anti-STING mAb (1:1000 dilution, #13647; Cell Signaling Technology), mouse anti-β-actin mAb (1:2000 dilution, #A5441; Sigma-Aldrich), rabbit anti-phospho-STING mAb (1:1000 dilution, #50907; Cell Signaling Technology) and mouse anti-GAPDH mAb (1:10000 dilution, #60004; Proteintech, Rosemont, IL, USA) were used for analysis.

### Colony formation assay

Breast cancer cells (1 × 10^3^ cells in 6-well plate) were treated with drugs for 15 days as described above. Colonies were then stained with Giemsa and measured using ImageJ software (NIH, Bethesda, MD, USA).

### Flow cytometry analysis

For cell cycle analysis and Annexin V staining assays, cells were treated with drugs for 9 days as described above. The cells were then stained with propidium iodide (Dojindo, Kumamoto, Japan) and an ApoScreen Annexin V Apoptosis Kit (Southern Biotech, Birmingham, AL, USA) according to the manufacturer’s instructions. Flow cytometric analysis was performed using a BD FACSCan II (BD Biosciences, Franklin Lakes, NJ, USA) with BD FACSDiva software (BD Biosciences). Data were analyzed using FlowJo software version 10 (FlowJo LLC, Ashland, OR, USA). To analyze cell surface human leukocyte antigen (HLA), cells were treated with drugs for 6 or 9 days as described above, after which they were labeled with mouse anti-HLA class I mAb (W6/32, ab22432, Abcam, Cambridge, UK) or mouse anti-HLA-DR mAb (L243, ab136320, Abcam). Goat anti-Mouse IgG (H + L) Secondary Antibody, PE (#M30004-1, Thermo Fisher Scientific, Waltham, MA, USA) was used as the secondary antibody. Flow cytometric analysis was then conducted using a BD FACSAria (BD Biosciences).

### Gene expression microarray analysis

Breast cancer cells were treated with drugs for 6 to 12 days as described above. Gene expression microarray analysis was then carried out using a SurePrint G3 Human GE microarray v2 (G4851; Agilent Technologies, Santa Clara, CA, USA). The microarray data were analyzed using GeneSpring GX version 14 (Agilent Technologies) and Gene Set Enrichment Analysis (GSEA; Broad Institute, Cambridge, MA, USA). The Gene Expression Omnibus accession number for the microarray data is GSE286942.

### Quantitative reverse transcription-PCR

Total RNA extraction, cDNA synthesis and quantitative reverse transcription-PCR (qRT-PCR) were performed as described previously [27]. Primer sequences and PCR product sizes are listed in Supplementary Table S1.

### Chromatin immunoprecipitation sequencing

Breast cancer cells were treated with drugs for 6 to 9 days as described above. Chromatin immunoprecipitation (ChIP) sequencing was performed as described previously using 0.2 µg of rabbit anti-trimethyl H3K4 Ab (#04-745, Sigma-Aldrich, St. Louis, MO, USA) [28]. Sequencing data were mapped to human genome hg38 using bowtie2, and peaks were called using MACS2. The genomic locations of the peaks were determined using ChIPpeakAnno [29]. Motif analysis was performed using HOMER v4.11. The NCBI SRA accession number for the ChIP-seq data is PRJNA1212475.

### Enzyme-linked immunosorbent assay

Breast cancer cells were treated with drugs for 6 days as described above. Enzyme-linked immunosorbent assays (ELISAs) for IFN-λ were performed using VeriKine-DIY Human Interferon Lambda 3/1/2 (IL-28B/29/28A) (PBL Assay Science, Piscataway, USA) according to the manufacturer’s instructions.

#### RNA sequencing

Breast cancer cells were treated with drugs for 6 days as described above. Total RNA was extracted using RNeasy Mini Kits (Qiagen) and genomic DNA was removed using a DNA-free DNA Removal Kit (Thermo Fisher Scientific) according to the manufacturers’ instructions. RNA sequencing (RNA-seq) was performed using an Illumina NovaSeq 6000 system (Illumina, San Diego, CA, USA) as described previously [30]. Mapping and quantification of the RNA-seq data were performed using the STAR-RSEM pipeline, after which the data were analyzed with Gene Spring GX version 14 (Agilent Technologies) and GSEA (Broad Institute). The NCBI SRA accession number for the RNA-seq data is PRJNA1212575. RNA-seq data from primary breast tumors and normal breast tissues in The Cancer Genome Atlas (TCGA) dataset were obtained using TCGAbiolinks.

#### CRISPR/Cas9 based gene knockout

For CRISPR/Cas9-based gene knockout in breast cancer cells, a lentiviral expression vector for Streptococcus pyogenes Cas9 and gRNA was used. Briefly, gRNA targeting STING1 or control gRNA was cloned into lentiCRISPR v2 (a gift from Dr. Feng Zhang, plasmid #52961, Addgene, Watertown, MA, USA). Thereafter, lentiviral particles expressing Cas9 and gRNA were produced using a Lenti-Pac HIV Expression Packaging Kit (GeneCopoeia, Rockville, MD, USA) according to the manufacturer’s instructions. After infection, cells were selected in culture medium containing 1.5 µg/mL of puromycin for 2 weeks. The gRNA sequences are listed in Supplementary Table S2. The efficacy of the STING1 knockout was confirmed by western blotting.

### Statistical analysis

Quantitative values were analyzed using Student’s t-test or two-way ANOVA. Results of cell viability assays and qRT-PCR were analyzed using Student’s t-test or two-way ANOVA. Each analyzed dataset was derived from at least three independent experiments because statistical significance was observed using the indicated sample sizes. Values of *P* < 0.05 (two-sided) were considered significant. Data were analyzed using GraphPad Prism 5 (GraphPad Software, Boston, MA, USA).

## Results

### Anti-tumor effects of DOT1L inhibitors in breast cancer cells

**Fig. 1:**
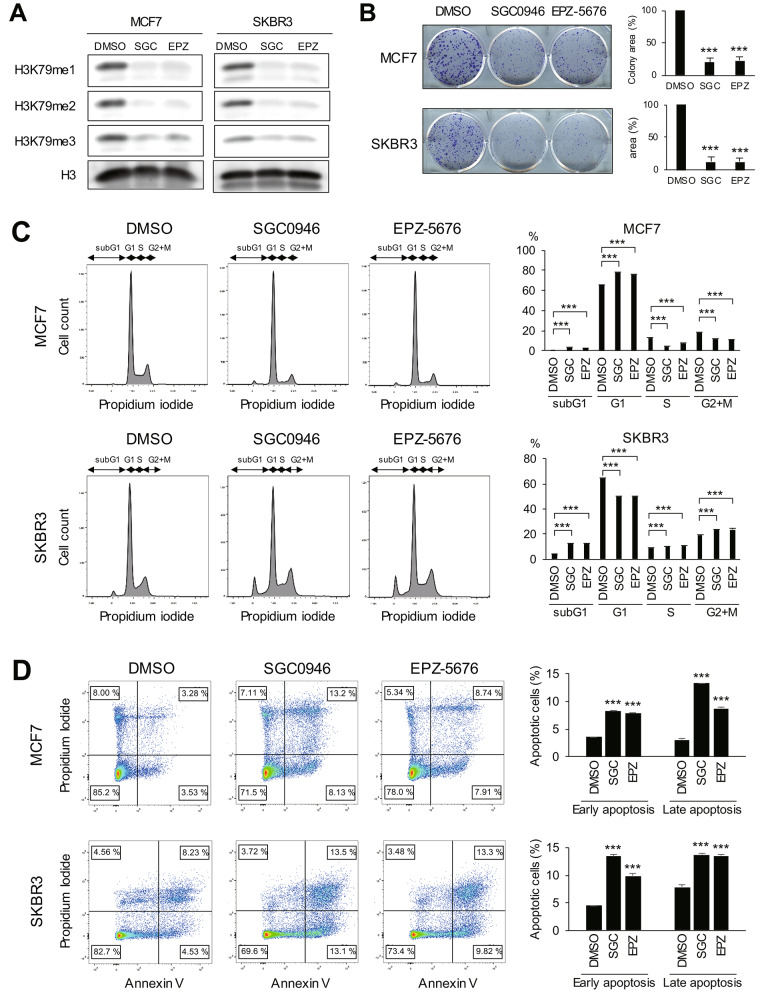
The anti-tumor effects of DOT1L inhibitors in breast cancer cells. **A** Western blot analysis of H3K79me1, H3K79me2 and H3K79me3 in MCF7 and SKBR3 cells treated for 12 days with DMSO, SGC0946 (SGC) or EPZ-5676 (EPZ). Total histone H3 is shown as an endogenous control. **B** Colony formation assays in breast cancer cells treated with DMSO or the indicated DOT1L inhibitor. Representative results are shown on the left, and summarized results are on the right. (n = 3). **C**, **D** Cell cycle (**C**) and apoptosis (**D**) analyses in breast cancer cells treated for 9 days with DMSO or the indicated DOT1L inhibitor. Representative results are shown on the left, and summarized results are on the right. (n = 3). Error bars represent SDs. ***P < 0.001

We first analyzed the expression of DOT1L in primary breast cancer tissues using RNA-seq data from the TCGA-BRCA dataset. We found that DOT1L expression levels were generally elevated across breast cancer subtypes, although the difference between HER2-positive tumors and normal tissues was not statistically significant (Supplementary Fig. S1). To confirm the anti-tumor effects of DOT1L inhibitors (SGC0946, EPZ-5676) in breast cancer, we used the MCF7 (ER-positive, HER2-negative) and SKBR3 (ER-negative, HER2-positive) cell lines for the analysis. We detected significantly lower levels of histone H3 K79 mono- (H3K79me1), di- (H3K79me2) and trimethylation (H3K79me3) in breast cancer cells treated with a DOT1L inhibitor (Fig. 1A). In addition, DOT1L inhibitors significantly suppressed colony formation in both cell lines (Fig. 1B). Cell cycle analysis showed that, in MCF7 cells, DOT1L inhibition induced G1 arrest and reduced S phase populations (Fig. 1C). Induction of G1 arrest by DOT1L inhibition was not apparent in SKBR3 cells, but a significant increase in the sub-G1 population was observed (Fig. 1C). Flow cytometric analysis further confirmed the induction of apoptosis by the DOT1L inhibitors in both cell lines (Fig. 1D). These results suggest that DOT1L inhibitors exert anti-tumor effects in both ER-positive/HER2-negative and HER2-positive/ER-negative breast cancer cells.

### DOT1L inhibition activates interferon signaling and immune responses in breast cancer cells

**Fig. 2:**
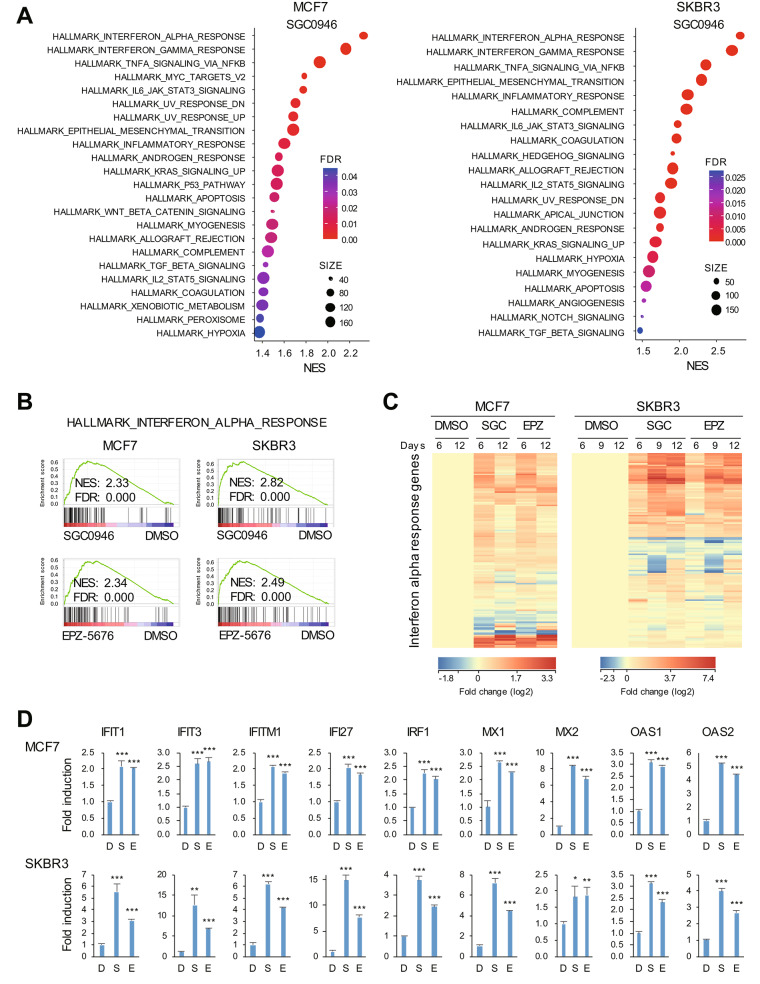
DOT1L inhibition activates interferon (IFN) signaling in breast cancer cells. **A** Gene expression microarray analysis in MCF7 (left) and SKBR3 (right) cells treated for 6 days with DMSO or SGC0946. Summarized results of GSEA using genes upregulated by SGC0946 are shown. NES, normalized enrichment score; FDR, false discovery rate. **B** Results of GSEA of the hallmark IFN-α response gene set in breast cancer cells treated for 6 days with the indicated DOT1L inhibitor. **C** Heatmaps showing the microarray data of the hallmark IFN-α response genes in breast cancer cells treated with DMSO, SGC0946 (SGC) or EPZ-5676 (EPZ) for indicated periods. **D** qRT-PCR analysis of the indicated IFN-related genes (IRGs) in breast cancer cells treated for 6 days with DMSO (D), SGC0946 (S) or EPZ-5676 (E). (n = 3). Error bars represent SDs. *P < 0.05, **P < 0.01, ***P < 0.001

To unravel the mechanism underlying the anti-tumor effect of DOT1L inhibition, we performed a gene expression microarray analysis with MCF7 and SKBR3 cells treated with a DOT1L inhibitor for 6 days. Consistent with earlier reports, GSEA revealed significant suppression of estrogen signaling by the DOT1L inhibitors in MCF7 cells (Supplementary Fig. S2A, B). By contrast, oxidative phosphorylation, MYC target genes and E2F targets were all strongly suppressed by DOT1L inhibition in SKBR3 cells (Supplementary Fig. S2A). Notably, GSEA revealed that DOT1L inhibition significantly activated IFN signaling in both MCF7 and SKBR3 cells (Fig. 2A, B, Supplementary Fig. 3A, B) and that effect was also confirmed by gene ontology and pathway analyses (Supplementary Fig. S4, S5). When we then extended our microarray analysis to cells treated with the drugs for up to 12 days, we observed persistent upregulation of IFN response genes during the treatment period (Fig. 2C, Supplementary Fig. S3C). qRT-PCR analysis of representative IFN-related genes (IRGs) confirmed robust upregulation by the drug treatment in both cell lines (Fig. 2D). These microarray and qRT-PCR analyses demonstrated that the peak induction of IRG expression in MCF7 cells occurred 6 days after the start of the drug treatment, whereas in SKBR3 cells, the upregulation was observed 9 days after treatment initiation (Fig. 2D, Supplementary Fig. S3D).

**Fig. 3:**
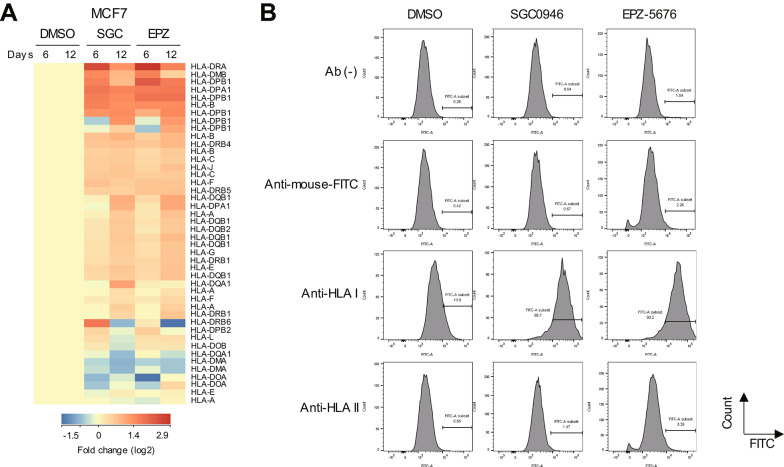
DOT1L inhibition upregulates HLA expression in breast cancer cells. **A** Heatmap showing microarray data of HLA genes in MCF7 cells treated with DMSO, SGC0946 (SGC) or EPZ-5676 (EPZ) for indicated periods. **B** Flow cytometry analysis showing cell surface expression of HLA class I and II molecules in MCF7 cell lines treated with indicated agents for 6 days

Pathway analysis of the microarray data suggested that DOT1L inhibition also upregulates genes related to class I MHC-mediated antigen processing and presentation (Supplementary Fig. S4, S5). As shown in Fig. 3A, our microarray analysis revealed that a number of HLA-related genes were upregulated by the drug treatment in both cell lines (Fig. 3A and data not shown). We therefore analyzed expression of cell surface HLA molecules in MCF7 cells, which revealed increased levels of HLA class I expression in cells upon drug treatment (Fig. 3B, Supplementary Fig. S6). Collectively, these results suggest that DOT1L inhibition activates tumor intrinsic innate immune responses in breast cancer cells.

To assess whether DOT1L inhibition activates immune responses against breast cancer cells, we co-cultured PBMCs, including lymphocytes, with breast cancer cells pre-treated with a DOT1L inhibitor (Supplementary Fig. S7A, S7B). qRT-PCR analysis revealed significantly increased expression of lymphotoxin alpha (LTA) and IFN-γ (IFNG) in PBMCs (Supplementary Fig. S7C). In contrast, indirect co-culture of PBMCs with pre-treated breast cancer cells did not upregulate these genes (Supplementary Fig. S7D). These results suggest that direct interaction between lymphocytes and DOT1L inhibitor-treated breast cancer cells may have activated the allogeneic lymphocytes.

### DOT1L inhibition activates IFN-Stat1 signaling in breast cancer cells

**Fig. 4:**
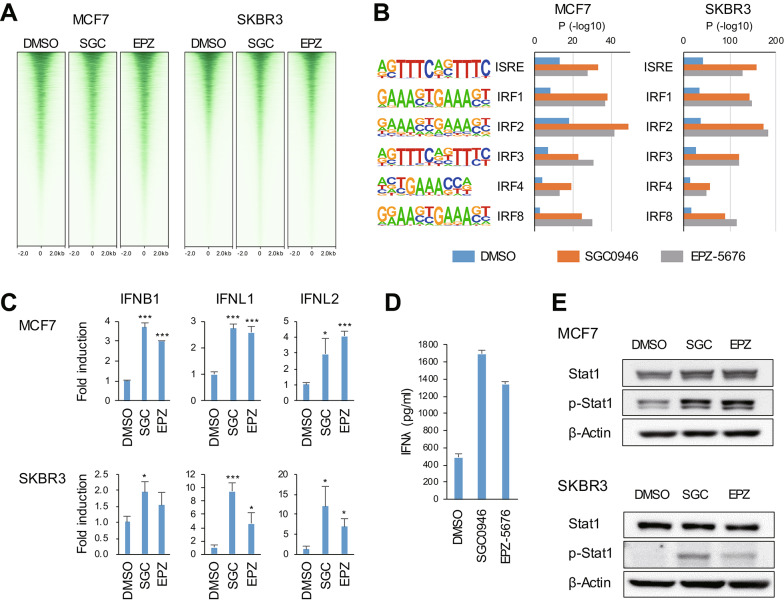
DOT1L inhibition activates IFN-Stat1 signaling in breast cancer cells. **A** Heatmaps showing histone H3 lysine 4 trimethylation (H3K4me3) peaks detected by ChIP-seq analysis in MCF7 and SKBR3 cells treated for 6 days with DMSO, SGC0946 (SGC) or EPZ-5676 (EPZ). **B** Motif analysis of H3K4me3 peaks in (**A**) in breast cancer cells treated as indicated. **C** qRT-PCR analysis of the indicated IFN genes in MCF7 cells treated for 6 days and SKBR3 cells treated for 9 days with the indicated agents. **D** ELISA of IFN-λ in MCF7 cells treated for 6 days with the indicated agents. **E** Western blot analysis of total and phosphorylated Stat1 in MCF7 cells treated for 6 days and SKBR3 cells treated for 9 days with indicated agents. Error bars represent SDs. *P < 0.05, ***P < 0.001

We next analyzed the effect of DOT1L inhibition on chromatin status in breast cancer cells. ChIP-seq analysis of histone H3 lysine 4 trimethylation (H3K4me3), a marker of active transcription start sites (TSSs), revealed increased numbers of H3K4me3 peaks in breast cancer cells treated with a DOT1L inhibitor (Fig. 4A, Supplementary Fig. S8A). Motif analysis demonstrated that IFN-stimulated response elements (ISREs) were significantly enriched around the H3K4me3 peaks in breast cancer cells treated with a DOT1L inhibitor (Fig. 4B, Supplementary Fig. S8B). We next performed qRT-PCR analysis of IFN genes and found that the drug treatment upregulated IFNB1, IFNL1 and IFNL2 in both cell lines (Fig. 4C, Supplementary Fig. S9). Induction of IFN-λ protein in MCF7 cells was further confirmed by ELISA (Fig. 4D). Western blot analysis demonstrated that DOT1L inhibition also upregulated levels of phosphorylated Stat1 (p-Stat1) in both cell lines (Fig. 4E). These results suggest that DOT1L inhibition activates IFN-Stat1 signaling in breast cancer cells.

### The STING pathway is involved in the activation of IFN signaling in breast cancer cells

**Fig. 5:**
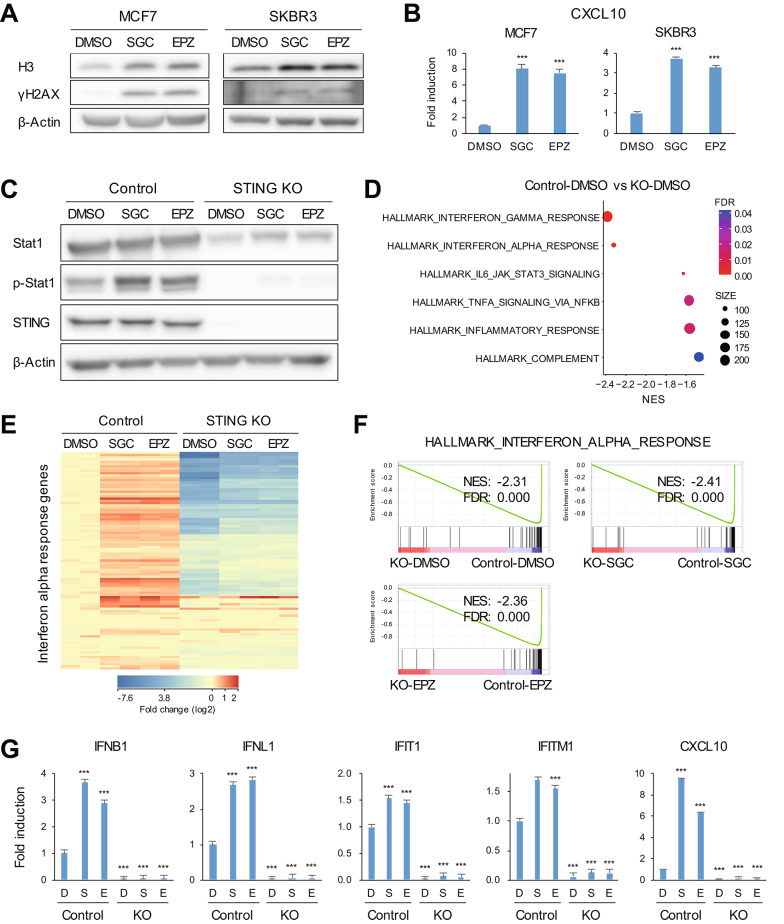
The STING pathway is involved in the DOT1L inhibition-induced IFN signaling in breast cancer cells. **A** Western blot analysis of histone H3 and γH2AX in the cytoplasm of MCF7 and SKBR3 cells treated for 6 days with DMSO, SGC0946 (SGC) or EPZ-5676 (EPZ). **B** qRT-PCR of CXCL10 in breast cancer cells treated for 6 days with the indicated agents. (n = 3). **C** Western blot analysis of total and phosphorylated Stat1 and STING in control and STING knockout (KO) MCF7 cells treated for 6 days with the indicated agents. **D** Results of RNA-seq analysis in control and STING KO MCF7 cells. Summarized results of GSEA of the indicated gene sets using genes downregulated in KO cells are shown. **E** Heatmap showing expression levels of the hallmark IFN-α response genes in control and STING KO MCF7 cells treated for 6 days with the indicated agents. **F** GSEA of the hallmark IFN-α response gene set in control and STING KO MCF7 cells treated for 6 days with the indicated agents. **G** qRT-PCR analysis of the indicated IFN genes and IRGs in control and STING KO MCF7 cells treated for 6 days with DMSO (D), SGC0946 (S) or EPZ-5676 (E). (n = 3). Error bars represent SDs. ***P < 0.001

Earlier studies have shown that DOT1L promotes DNA repair by recruiting factors associated with nucleotide excision repair and homologous recombination [31–33]. We therefore analyzed cytoplasmic DNA as a DNA damage marker in breast cancer cells. Indicative of the increased levels of cytoplasmic DNA, we detected elevated levels of histone H3 in the cytoplasm of breast cancer cells treated with a DOT1L inhibitor (Fig. 5A, Supplementary Fig. S10A). Likewise, we found increased levels of γH2AX, a marker of DNA damage, in the cytoplasmic and nuclear fractions of breast cancer cells treated with a DOT1L inhibitor (Fig. 5A, Supplementary Fig. S10A, B). We also found that DOT1L inhibition upregulated a DNA damage-response cytokine, CXCL10 (Fig. 5B, Supplementary Fig. S10C) [34]. These results suggest that DOT1L inhibition induces DNA damage in breast cancer cells. In addition, fluorescence staining of double-stranded DNA (dsDNA) revealed cytoplasmic DNA foci in breast cancer cells treated with a DOT1L inhibitor, indicating increased levels of cytosolic DNA upon DOT1L inhibition (Supplementary Fig. S11). Moreover, DOT1L inhibition increased the levels of phosphorylated STING in breast cancer cells, indicating activation of the cytosolic DNA sensor pathway (Supplementary Fig. S10D).

To test whether the cytosolic DNA sensor pathway is involved in DOT1L inhibition-induced IFN-Stat1 signaling, we established MCF7 cells with CRISPR/Cas9-mediated genetic knockout of *STING1*, the gene encoding STING. We observed a marked decrease in the levels of total and p-Stat1 in STING knockout cells as compared to wild-type cells (Fig. 5C). Weak upregulation of total Stat1 was observed in the knockout cells treated with a DOT1L inhibitor, while the levels were lower than those in wild-type cells (Fig. 5C). Moreover, induction of p-Stat1 by DOT1L inhibition was strikingly attenuated in the knockout cells (Fig. 5C). Subsequent transcriptome analysis in control and STING knockout cells revealed that basal levels of IFN response genes were significantly downregulated in the knockout cells (Fig. 5D, Supplementary Fig. S12A). Treatment with a DOT1L inhibitor marginally upregulated IFN response genes in the knockout cells, though the levels were strikingly lower than those in the control cells (Fig. 5E, F, Supplementary Fig. S12B, S12C). qRT-PCR analysis confirmed the decreased expression of IFN genes and representative IRGs in the knockout cells (Fig. 5G, Supplementary Fig. S12D). These results suggest the STING pathway plays an essential role in the DOT1L inhibition-induced IFN-Stat1 signaling in breast cancer cells.

### Loss of STING attenuates the anti-tumor effect of DOT1L Inhibition in breast cancer cells

**Fig. 6:**
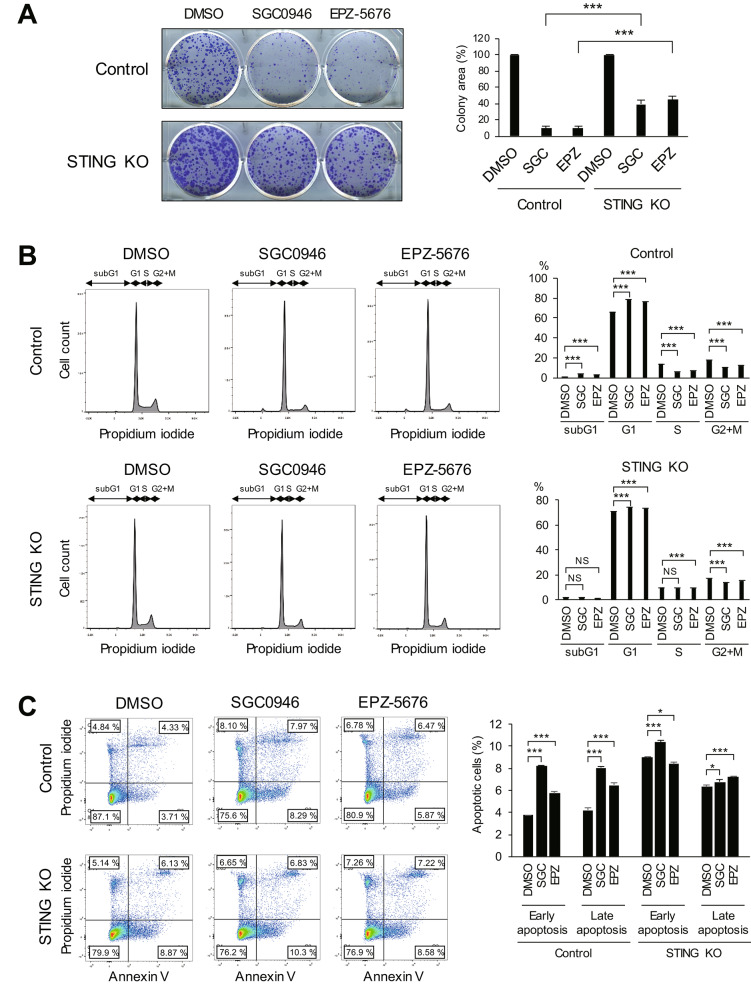
The STING pathway contributes to the anti-tumor effect of DOT1L inhibition in breast cancer cells. **A** Colony formation assays in control and STING knockout (KO) MCF7 cells treated with DMSO, SGC0946 or EPZ-5676. Representative results are shown on the left, and summarized results are on the right. (n = 3). **B**, **C** Cell cycle (**B**) and apoptosis (**C**) assays in control and STING KO MCF7 cells treated for 9 days with DMSO, SGC0946 (SGC) or EPZ-5676 (EPZ). Representative results are shown on the left, and summarized results are on the right. NS, not significant; ***P < 0.001

We next assessed whether the STING pathway is involved in the anti-tumor effect of DOT1L inhibition. Colony formation assays revealed that the growth suppressive effects of DOT1L inhibitors were attenuated in the STING knockout cells (Fig. 6A). Cell cycle analysis showed that the G1 arrest and the increases in sub-G1 populations induced by DOT1L inhibition were attenuated in the knockout cells (Fig. 6B). Flow cytometric analysis revealed that apoptosis induction was also attenuated in the knockout cells (Fig. 6C). Notably, in the absence of DOT1L inhibition, STING knockout cells exhibited higher levels of apoptosis than control cells, suggesting that basal levels of STING expression may inhibit apoptosis in breast cancer cells (Fig. 6C). These results suggest that the STING pathway contributes to the anti-tumor effect of DOT1L inhibition in breast cancer.

### DOT1L inhibition suppresses ERBB2 expression in HER2-positive breast cancer cells

**Fig. 7:**
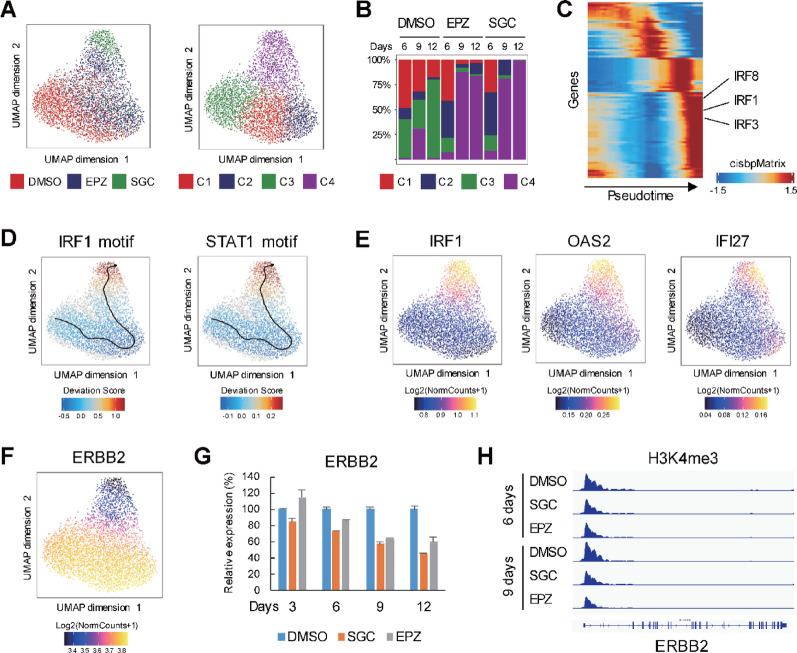
Single-cell ATAC-seq (scATAC-seq) analysis in SKBR3 cells treated with a DOT1L inhibitor. **A** Uniform manifold approximation and projection (UMAP) of the scATAC-seq data from SKBR3 cells treated with DMSO, SGC0946 (SGC) or EPZ-5676 (EPZ). Results of unsupervised clustering are shown on the right. **B** Proportions of the respective clusters in cells treated as indicated. **C** Summarized results of a pseudotime analysis of motifs identified by scATAC-seq. **D** Results of a pseudotime trajectory analysis of IRF1 and STAT1 motifs. **E** Gene scores of representative IRGs. **F** Gene scores of ERBB2 gene. **G** Results of a qRT-PCR analysis of ERBB2 in SKBR3 cells treated with the indicated agents. (n = 3). **H** Results of ChIP-seq analysis showing levels of H3K4me3 at the ERBB2 gene in SKBR3 cells treated as indicated

The results summarized above suggest that DOT1L inhibition exerts its anti-tumor effect in MCF7 cells by inhibiting estrogen signaling and inducing IFN signaling. To further clarify the anti-tumor mechanism of DOT1L inhibition in HER2-positive breast cancer cells, we performed Single-cell Assay for Transposase-Accessible Chromatin sequencing (scATAC-seq) analysis in SKBR3 cells treated with DMSO, SGC0946 or EPZ-5676 for 6 to 12 days (Supplementary Fig. S13). Unsupervised clustering analysis of the scATAC-seq data suggested that the cells could be divided into 4 clusters (Fig. 7A). The proportions of the clusters in the respective treatment conditions showed that the majority of cells treated for longer periods (9 and 12 days) were categorized into cluster 4 (Fig. 7B). Pseudotime analysis suggested that motifs associated with IFN signaling were enriched in open chromatin regions in cells after extended treatment, which is consistent with the results of our transcriptome and epigenome analyses (Fig. 7C, D). Gene scores calculated using the scATAC-seq data also suggested elevated expression of representative IRGs in cells treated with a DOT1L inhibitor, which further confirmed the reliability of our scATAC-seq analysis (Fig. 7E). Notably, the scATAC-seq data demonstrated that the chromatin structure of ERBB2, the gene encoding HER2, was closed in cells after extended treatment with a DOT1L inhibitor (Fig. 7F), and qRT-PCR analysis confirmed that DOT1L inhibitors suppressed ERBB2 expression in a time-dependent manner (Fig. 7G). Analysis of the ChIP-seq data demonstrated that suppression of H3K4me3 levels at the transcription start site of ERBB2 by DOT1L inhibitors is also time-dependent (Fig. 7H). These results suggest the anti-tumor effect of DOT1L inhibition in HER2-positive breast cancer cells is mediated, at least in part, through downregulation of ERBB2.

## Discussion

In the present study, we identified a novel mechanism by which DOT1L inhibition exerts its anti-tumor effect in breast cancer. We found that DOT1L inhibition induces DNA damage, which leads to activation of IFN-Stat1 signaling in both ER-positive/HER2-negative and HER2-positive/ER-negative breast cancer cells. These findings in the present study are consistent with our earlier observation that DOT1L inhibition upregulates a number of IRGs in multiple myeloma cells [15]. Genetic ablation of STING in breast cancer cells attenuated the activation of IFN signaling and growth suppression elicited upon DOT1L inhibition, suggesting the STING pathway contributes to the anti-breast cancer effect of DOT1L inhibition. We also found that DOT1L inhibition downregulates ERBB2 expression in HER2-positive breast cancer cells, which may contribute to the anti-tumor effect. In addition, our observation that DOT1L inhibition upregulates HLA class I expression in breast cancer cells suggests it may sensitize cancer cells to tumor immunotherapy.

In recent years, a number of studies have shown that DOT1L inhibition exerts anti-breast cancer effects through multiple mechanisms. Cho et al.. showed that in breast cancer cells, DOT1L cooperates with c-Myc and p300 acetyltransferase to activate a series of EMT-related genes, including SNAIL, ZEB1 and ZEB2, and that the DOT1L-c-Myc-p300 complex enhances the CSC properties [22]. Nassa et al.. demonstrated that DOT1L is a novel cofactor of ERα and that DOT1L inhibition suppresses proliferation of antiestrogen (AE)-sensitive and AE-resistant breast cancer cells [23]. Salvati et al.. detected an association between DOT1L and menin and revealed that they co-regulate target genes involved in estrogen, p53, HIF1α and death receptor signaling in breast cancer cells [35]. They also showed that inhibiting DOT1L and menin synergistically inhibits proliferation of AE-sensitive and AE-resistant breast cancer cells [35]. Taken together with these observations, our results demonstrate that the anti-breast cancer effects of DOT1L inhibition are mediated via modulation of a variety of biological processes, including growth and apoptosis signaling, EMT, CSC activity, and innate immune signaling.

It is now apparent that DOT1L plays an essential role in the DNA damage response. An earlier study showed that H3K79 methylation is required for recruitment of 53BP1, a DNA double strand break (DSB) sensor protein, to DSB sites and that suppression of DOT1L inhibits 53BP1 recruitment [31]. A subsequent study showed that loss of 53BP1 and DOT1L induces a striking DNA damage-resistant phenotype in chicken DT40 cells [32]. In addition, Kari et al. showed that in rectal cancer cells, DOT1L is required for a proper DNA damage response following DSBs by regulating the phosphorylation of γH2AX, and that DOT1L is also required for homologous recombination-mediated DNA DSB repair [36]. They also showed that DOT1L inhibition upregulates the sensitivity of rectal cancer cells to DNA damaging agents [36]. Another recent study reported that DOT1L plays a critical role in DNA repair by initiating recruitment of RAP80 and BRCA1 to DSB sites [37]. Recently, we reported that DOT1L inhibition induces DNA damage and activates IFN signaling in multiple myeloma (MM) cells [26]. Knockout of *STING1* attenuated the induction of IRGs as well as the anti-tumor effect of DOT1L inhibition in MM cells [26]. Taken together with these reports, our findings suggest that DOT1L inhibition disrupts DNA damage responses, which leads to upregulation of cytosolic DNA and activation of the STING pathway in breast cancer cells.

Several recent studies have shown that the cGAS-STING pathway is involved in the therapeutic responses of breast cancer. For instance, Pantelidou et al.. reported that the CD8 + T-cell infiltration elicited by PARP inhibition is mediated through the STING/TBK1/IRF3 pathway in breast cancer [38]. Experiments using breast cancer patient-derived xenografts revealed that paclitaxel treatment activates type I IFN and TNFα and that a cGAS-STING-dependent apoptotic effect is required for the paclitaxel response in vivo [39]. Another recent study showed that a nuclear receptor, NR1D1, enhances the antitumor CD8 + T cell response to breast cancer by promoting DNA damage-induced accumulation of cytosolic DNA fragments, which leads to activation of cGAS-STING signaling and downstream type I IFN signaling [40]. Parks et al.. reported that activation of a cGAS-STING-mediated immune response could be a predictive marker of a neoadjuvant chemotherapy response in breast cancer [41]. Activation of the cGAS-STING pathway also reportedly correlates with genomic instability and better responses to immunotherapy in breast cancer patients [42]. And Zhang et al.. demonstrated that attenuation of cGAS-STING signaling is associated with resistance to endocrine therapy in ER-positive breast cancer [43]. In contrast to those reports, other studies have described tumor-promoting actions of cGAS-STING in breast cancer. Cheradame et al.. discovered a non-canonical tumor-promoting action whereby STING protects breast cancer cells from DNA instability [44]. In addition, Hong et al.. reported that the cGAS-STING pathway promotes survival of triple negative breast cancer (TNBC) cells with chromosomal instability by activating IL6-STAT3 signaling [45]. Our results support the hypothesis that DOT1L inhibition suppresses breast cancer cell proliferation by activating STING and types I and III IFN signaling, but further study will be necessary to clarify the effect of DOT1L inhibition in TNBC cells with chromosomal instability.

There are several limitations in this study. First, although our results suggest that DOT1L inhibition activates innate immune responses in breast cancer cells, we did not assess the effect of DOT1L inhibition on tumor immune responses using an in vivo model. Second, our study did not clarify whether DOT1L inhibition activates immune responses in TNBC cells. A recent study showed that DOT1L inhibition does not suppress proliferation of TNBC cells in vitro, but it decreases in vivo tumor growth by inhibiting CSC properties [24]. Further study will be necessary to determine whether DOT1L inhibition affects DNA damage and IFN signaling in TNBC CSCs.

## Conclusion

In conclusion, our findings show that DOT1L inhibition induces DNA damage and activates STING-mediated IFN signaling in breast cancer cells. We also found that DOT1L inhibition suppresses ERBB2 expression in HER2-positive breast cancer cells. This suggests that adding DOT1L inhibition may enhance the beneficial effects of chemotherapy and tumor immunotherapy. Further study to clarify the clinical usefulness of DOT1L inhibition in breast cancer is therefore warranted.

## Supplementary Information

Below is the link to the electronic supplementary material.

Supplementary Material 1

Supplementary Material 2

Supplementary Material 3

Supplementary Material 4

## Data Availability

The Gene Expression Omnibus accession number for the microarray data is GSE286942. ChIP-seq and RNA-seq data were deposited in the NCBI SRA (accession ID, PRJNA1212475, PRJNA1212575).

## Abbreviations

AE: Antiestrogen
ANOVA: Analysis of variance
ChIP-seq: Chromatin immunoprecipitation-sequencing
cGAS: Cyclic GMP-AMP synthase
CSC: Cancer stem cell
CXCL10: C-X-C motif chemokine ligand 10
DSB: Double strand break
DOT1L: Dot1-like
ELISA: Enzyme-linked immunosorbent assay
EMT: Epithelial-mesenchymal transition
ER: Estrogen receptor
ERBB2: Erb-B2 receptor tyrosine kinase 2
GO: Gene ontology
GSEA: Gene set enrichment analysis
HIF1α: Hypoxia-inducible factor 1 alpha
HLA: Human leukocyte antigen
IFN: Interferon
IRG: Interferon-related genes
ISRE: Interferon-stimulated response elements
LTA: Lymphotoxin alpha
qRT-PCR: Quantitative reverse transcription-polymerase chain reaction
RNA-seq: RNA sequencing
scATAC-seq: Single-cell Assay for Transposase-Accessible Chromatin-sequencing
STAT1: Signal transducer and activator of transcription 1
STING: Stimulator of interferon genes
TNBC: Triple-negative breast cancer
TSS: Transcription start site

## References

[1] Bray F, Laversanne M, Sung H, Ferlay J, Siegel RL, Soerjomataram I, et al. Global cancer statistics 2022: GLOBOCAN estimates of incidence and mortality worldwide for 36 cancers in 185 countries. CA Cancer J Clin. 2024;74(3):229–63. 38572751 10.3322/caac.21834

[2] Sorlie T, Perou CM, Tibshirani R, Aas T, Geisler S, Johnsen H, et al. Gene expression patterns of breast carcinomas distinguish tumor subclasses with clinical implications. Proc Natl Acad Sci U S A. 2001;98(19):10869–74. 11553815 10.1073/pnas.191367098 PMC58566

[3] Gradishar WJ, Moran MS, Abraham J, Aft R, Agnese D, Allison KH, et al. Breast Cancer, version 3.2022, NCCN clinical practice guidelines in oncology. J Natl Compr Canc Netw. 2022;20(6):691–722. 35714673 10.6004/jnccn.2022.0030

[4] Kang C, Syed YY. Atezolizumab (in combination with Nab-Paclitaxel): a review in advanced triple-negative breast cancer. Drugs. 2020;80(6):601–7. 32248356 10.1007/s40265-020-01295-y

[5] Laisne M, Lupien M, Vallot C. Epigenomic heterogeneity as a source of tumour evolution. Nat Rev Cancer. 2025;25(1):7–26. 39414948 10.1038/s41568-024-00757-9

[6] Zhao S, Allis CD, Wang GG. The language of chromatin modification in human cancers. Nat Rev Cancer. 2021;21(7):413–30. 34002060 10.1038/s41568-021-00357-x PMC10507815

[7] Nguyen AT, Zhang Y. The diverse functions of Dot1 and H3K79 methylation. Genes Dev. 2011;25(13):1345–58. 21724828 10.1101/gad.2057811 PMC3134078

[8] Jones B, Su H, Bhat A, Lei H, Bajko J, Hevi S, et al. The histone H3K79 methyltransferase Dot1L is essential for mammalian development and heterochromatin structure. PLoS Genet. 2008;4(9):e1000190. 18787701 10.1371/journal.pgen.1000190 PMC2527135

[9] Feng Q, Wang H, Ng HH, Erdjument-Bromage H, Tempst P, Struhl K, et al. Methylation of H3-lysine 79 is mediated by a new family of hmtases without a SET domain. Curr Biol. 2002;12(12):1052–8. 12123582 10.1016/s0960-9822(02)00901-6

[10] Cattaneo P, Kunderfranco P, Greco C, Guffanti A, Stirparo GG, Rusconi F, et al. DOT1L-mediated H3K79me2 modification critically regulates gene expression during cardiomyocyte differentiation. Cell Death Differ. 2016;23(4):555–64. 25526092 10.1038/cdd.2014.199 PMC4986629

[11] Jo SY, Granowicz EM, Maillard I, Thomas D, Hess JL. Requirement for Dot1l in murine postnatal hematopoiesis and leukemogenesis by MLL translocation. Blood. 2011;117(18):4759–68. 21398221 10.1182/blood-2010-12-327668 PMC3100687

[12] Bernt KM, Zhu N, Sinha AU, Vempati S, Faber J, Krivtsov AV, et al. MLL-rearranged leukemia is dependent on aberrant H3K79 methylation by DOT1L. Cancer Cell. 2011;20(1):66–78. 21741597 10.1016/j.ccr.2011.06.010 PMC3329803

[13] Daigle SR, Olhava EJ, Therkelsen CA, Majer CR, Sneeringer CJ, Song J, et al. Selective killing of mixed lineage leukemia cells by a potent small-molecule DOT1L inhibitor. Cancer Cell. 2011;20(1):53–65. 21741596 10.1016/j.ccr.2011.06.009 PMC4046888

[14] Daigle SR, Olhava EJ, Therkelsen CA, Basavapathruni A, Jin L, Boriack-Sjodin PA, et al. Potent inhibition of DOT1L as treatment of MLL-fusion leukemia. Blood. 2013;122(6):1017–25. 23801631 10.1182/blood-2013-04-497644 PMC3739029

[15] Ishiguro K, Kitajima H, Niinuma T, Ishida T, Maruyama R, Ikeda H, et al. DOT1L inhibition blocks multiple myeloma cell proliferation by suppressing IRF4-MYC signaling. Haematologica. 2019;104(1):155–65. 30171029 10.3324/haematol.2018.191262 PMC6312027

[16] Zhang X, Liu D, Li M, Cao C, Wan D, Xi B, et al. Prognostic and therapeutic value of disruptor of telomeric silencing-1-like (DOT1L) expression in patients with ovarian cancer. J Hematol Oncol. 2017;10(1):29. 28114995 10.1186/s13045-017-0400-8 PMC5259947

[17] Liu D, Zhang XX, Li MC, Cao CH, Wan DY, Xi BX, et al. C/EBPbeta enhances platinum resistance of ovarian cancer cells by reprogramming H3K79 methylation. Nat Commun. 2018;9(1):1739. 29712898 10.1038/s41467-018-03590-5 PMC5928165

[18] Wong M, Tee AEL, Milazzo G, Bell JL, Poulos RC, Atmadibrata B, et al. The histone methyltransferase DOT1L promotes neuroblastoma by regulating gene transcription. Cancer Res. 2017;77(9):2522–33. 28209620 10.1158/0008-5472.CAN-16-1663

[19] Yang L, Lei Q, Li L, Yang J, Dong Z, Cui H. Silencing or inhibition of H3K79 methyltransferase DOT1L induces cell cycle arrest by epigenetically modulating c-Myc expression in colorectal cancer. Clin Epigenetics. 2019;11(1):199. 31888761 10.1186/s13148-019-0778-y PMC6937672

[20] Vatapalli R, Sagar V, Rodriguez Y, Zhao JC, Unno K, Pamarthy S, et al. Histone methyltransferase DOT1L coordinates AR and MYC stability in prostate cancer. Nat Commun. 2020;11(1):4153. 32814769 10.1038/s41467-020-18013-7 PMC7438336

[21] Zhang L, Deng L, Chen F, Yao Y, Wu B, Wei L, et al. Inhibition of histone H3K79 methylation selectively inhibits proliferation, self-renewal and metastatic potential of breast cancer. Oncotarget. 2014;5(21):10665–77. 25359765 10.18632/oncotarget.2496 PMC4279401

[22] Cho MH, Park JH, Choi HJ, Park MK, Won HY, Park YJ, et al. DOT1L cooperates with the c-Myc-p300 complex to epigenetically derepress CDH1 transcription factors in breast cancer progression. Nat Commun. 2015;6:7821. 26199140 10.1038/ncomms8821 PMC4525167

[23] Nassa G, Salvati A, Tarallo R, Gigantino V, Alexandrova E, Memoli D, et al. Inhibition of histone methyltransferase DOT1L silences ERalpha gene and blocks proliferation of antiestrogen-resistant breast cancer cells. Sci Adv. 2019;5(2):eaav5590. 30775443 10.1126/sciadv.aav5590 PMC6365116

[24] Kurani H, Razavipour SF, Harikumar KB, Dunworth M, Ewald AJ, Nasir A, et al. DOT1L is a novel cancer stem cell target for triple-negative breast cancer. Clin Cancer Res. 2022;28(9):1948–65. 35135840 10.1158/1078-0432.CCR-21-1299 PMC9365344

[25] Suzuki H, Toyota M, Carraway H, Gabrielson E, Ohmura T, Fujikane T, et al. Frequent epigenetic inactivation of Wnt antagonist genes in breast cancer. Br J Cancer. 2008;98(6):1147–56. 18283316 10.1038/sj.bjc.6604259 PMC2275475

[26] Ishiguro K, Kitajima H, Niinuma T, Maruyama R, Tsukahara T, Hirohashi Y, et al. DOT1L inhibition reprograms innate immunity to potentiate immunomodulatory drug responses in multiple myeloma. Cancer Lett. 2025;631:217941. 40701319 10.1016/j.canlet.2025.217941

[27] Kitajima H, Maruyama R, Niinuma T, Yamamoto E, Takasawa A, Takasawa K, et al. TM4SF1-AS1 inhibits apoptosis by promoting stress granule formation in cancer cells. Cell Death Dis. 2023;14(7):424. 37443145 10.1038/s41419-023-05953-3 PMC10345132

[28] Ishiguro K, Kitajima H, Niinuma T, Maruyama R, Nishiyama N, Ohtani H, et al. Dual EZH2 and G9a inhibition suppresses multiple myeloma cell proliferation by regulating the interferon signal and IRF4-MYC axis. Cell Death Discov. 2021;7(1):7. 33436557 10.1038/s41420-020-00400-0 PMC7803977

[29] Zhu LJ, Gazin C, Lawson ND, Pages H, Lin SM, Lapointe DS, et al. ChIPpeakAnno: a bioconductor package to annotate ChIP-seq and ChIP-chip data. BMC Bioinform. 2010;11:237. 10.1186/1471-2105-11-237 PMC3098059 20459804

[30] Nishiyama H, Niinuma T, Kitajima H, Ishiguro K, Yamamoto E, Sudo G et al. HOXA11-As promotes lymph node metastasis through regulation of IFNL and HMGB family genes in pancreatic cancer. Int J Mol Sci. 2024;25(23). 10.3390/ijms252312920 PMC11641524 39684631

[31] Huyen Y, Zgheib O, Ditullio RA Jr., Gorgoulis VG, Zacharatos P, Petty TJ, et al. Methylated lysine 79 of histone H3 targets 53BP1 to DNA double-strand breaks. Nature. 2004;432(7015):406–11. 15525939 10.1038/nature03114

[32] FitzGerald J, Moureau S, Drogaris P, O’Connell E, Abshiru N, Verreault A, et al. Regulation of the DNA damage response and gene expression by the Dot1L histone methyltransferase and the 53Bp1 tumour suppressor. PLoS ONE. 2011;6(2):e14714. 21383990 10.1371/journal.pone.0014714 PMC3044716

[33] Wakeman TP, Wang Q, Feng J, Wang XF. Bat3 facilitates H3K79 dimethylation by DOT1L and promotes DNA damage-induced 53BP1 foci at G1/G2 cell-cycle phases. EMBO J. 2012;31(9):2169–81. 22373577 10.1038/emboj.2012.50 PMC3343460

[34] Parkes EE, Walker SM, Taggart LE, McCabe N, Knight LA, Wilkinson R et al. Activation of STING-dependent innate immune signaling by S-Phase-Specific DNA damage in breast cancer. J Natl Cancer Inst. 2017;109(1). 10.1093/jnci/djw199 PMC5441301 27707838

[35] Salvati A, Melone V, Sellitto A, Rizzo F, Tarallo R, Nyman TA, et al. Combinatorial targeting of a chromatin complex comprising Dot1L, Menin and the tyrosine kinase BAZ1B reveals a new therapeutic vulnerability of endocrine therapy-resistant breast cancer. Breast Cancer Res. 2022;24(1):52. 35850772 10.1186/s13058-022-01547-7 PMC9290241

[36] Kari V, Raul SK, Henck JM, Kitz J, Kramer F, Kosinsky RL, et al. The histone methyltransferase DOT1L is required for proper DNA damage response, DNA repair, and modulates chemotherapy responsiveness. Clin Epigenetics. 2019;11(1):4. 30616689 10.1186/s13148-018-0601-1 PMC6323691

[37] Tang H, Lu YF, Zeng R, Liu C, Shu Y, Wu Y, et al. DOT1L-mediated RAP80 methylation promotes BRCA1 recruitment to elicit DNA repair. Proc Natl Acad Sci U S A. 2024;121(35):e2320804121. 39172790 10.1073/pnas.2320804121 PMC11363320

[38] Pantelidou C, Sonzogni O, De Oliveria Taveira M, Mehta AK, Kothari A, Wang D, et al. PARP inhibitor efficacy depends on CD8(+) T-cell recruitment via intratumoral STING pathway activation in BRCA-deficient models of triple-negative breast cancer. Cancer Discov. 2019;9(6):722–37. 31015319 10.1158/2159-8290.CD-18-1218 PMC6548644

[39] Lohard S, Bourgeois N, Maillet L, Gautier F, Fetiveau A, Lasla H, et al. STING-dependent paracriny shapes apoptotic priming of breast tumors in response to anti-mitotic treatment. Nat Commun. 2020;11(1):259. 31937780 10.1038/s41467-019-13689-y PMC6959316

[40] Ka NL, Park MK, Kim SS, Jeon Y, Hwang S, Kim SM, et al. NR1D1 stimulates antitumor immune responses in breast cancer by activating cGAS-STING signaling. Cancer Res. 2023;83(18):3045–58. 37395684 10.1158/0008-5472.CAN-23-0329 PMC10538367

[41] Parkes EE, Savage KI, Lioe T, Boyd C, Halliday S, Walker SM, et al. Activation of a cGAS-STING-mediated immune response predicts response to neoadjuvant chemotherapy in early breast cancer. Br J Cancer. 2022;126(2):247–58. 34728791 10.1038/s41416-021-01599-0 PMC8770594

[42] Chen M, Yu S, van der Sluis T, Zwager MC, Schroder CP, van der Vegt B, et al. cGAS-STING pathway expression correlates with genomic instability and immune cell infiltration in breast cancer. NPJ Breast Cancer. 2024;10(1):1. 38167507 10.1038/s41523-023-00609-z PMC10761738

[43] Zhang KM, Zhao DC, Li ZY, Wang Y, Liu JN, Du T, et al. Inactivated cGAS-STING signaling facilitates endocrine resistance by forming a positive feedback loop with AKT kinase in ER + HER2- breast cancer. Adv Sci (Weinh). 2024;11(35):e2403592. 39023171 10.1002/advs.202403592 PMC11425221

[44] Cheradame L, Guerrera IC, Gaston J, Schmitt A, Jung V, Goudin N, et al. STING protects breast cancer cells from intrinsic and genotoxic-induced DNA instability via a non-canonical, cell-autonomous pathway. Oncogene. 2021;40(49):6627–40. 34625708 10.1038/s41388-021-02037-4

[45] Hong C, Schubert M, Tijhuis AE, Requesens M, Roorda M, van den Brink A, et al. cGAS-STING drives the IL-6-dependent survival of chromosomally instable cancers. Nature. 2022;607(7918):366–73. 35705809 10.1038/s41586-022-04847-2

