# Supplementary material for: DOT1L inhibition exerts the anti-tumor effect by activating interferon signaling in breast cancer cells
Source: Clin Epigenetics. 2025 Nov 26;17:201. doi: 10.1186/s13148-025-02017-5 (PMC12659069; doi:10.1186/s13148-025-02017-5)
Supplement: Supplementary file 1 — Supplementary Material 1 [file 13148_2025_2017_MOESM1_ESM.pdf]

Supplementary Figure S1

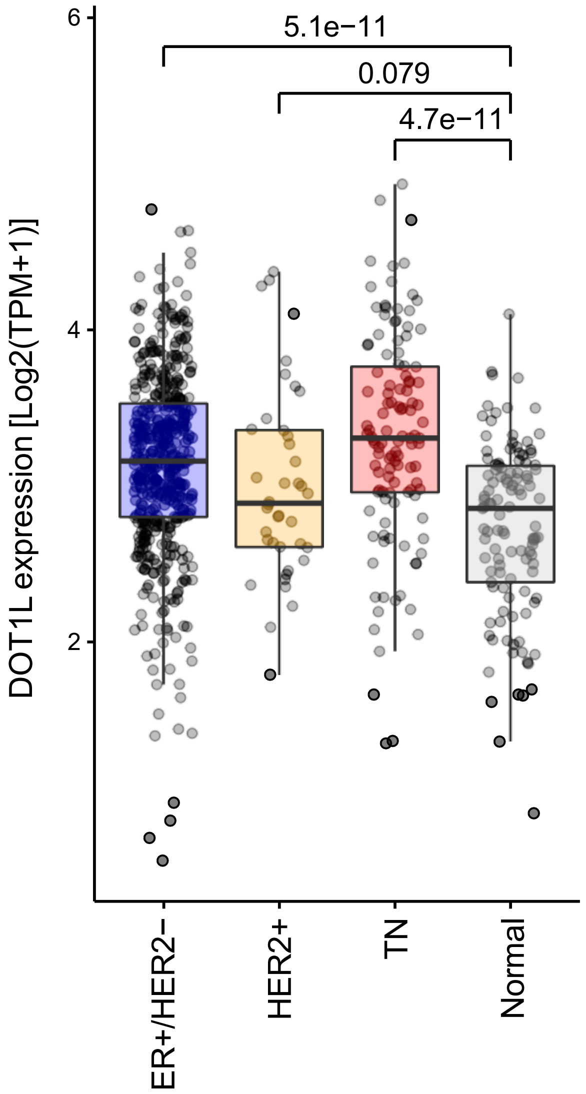

Supplementary Figure S1

Levels of DOT1L expression in primary breast cancer tissues and normal breast tissues based on RNA-seq data from The Cancer Genome Atlas (TCGA)-BRCA dataset. TN, triple negative.

Supplementary Figure S2

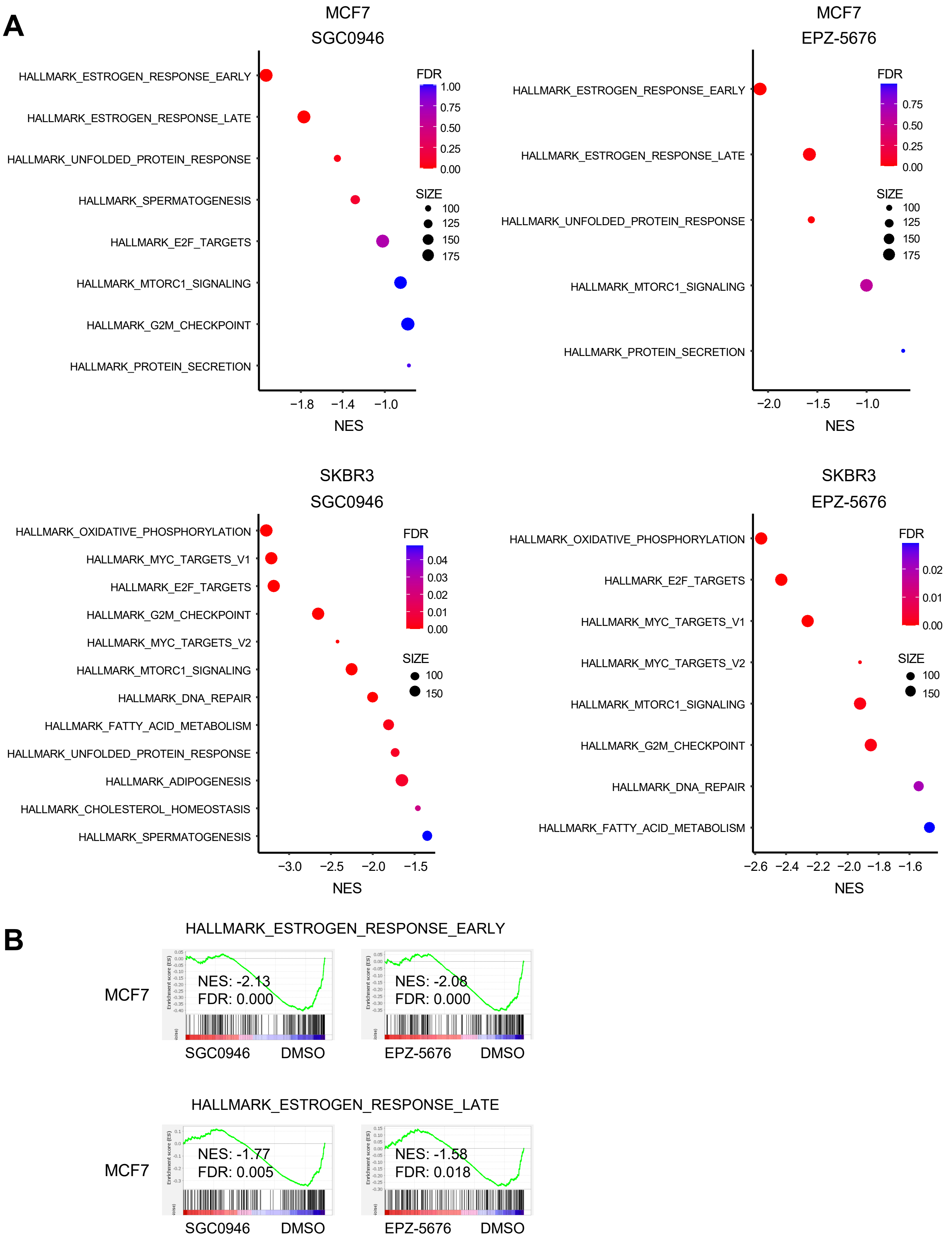

Supplementary Figure S2

Effects of DOT1L inhibition on gene expression profiles in breast cancer cells. (A) Gene expression microarray analysis in MCF7 or SKBR3 cells treated for 6 days with DMSO, SGC0946 or EPZ-5676. Summarized results of GSEA using genes downregulated by the indicated DOT1L inhibitor are shown. NES, normalized enrichment score; FDR, false discovery rate. (B) GSEA of the estrogen response early (upper) and late (lower) gene sets in MCF7 cells treated for 6 days with the indicated DOT1L inhibitor.

Supplementary Figure S3

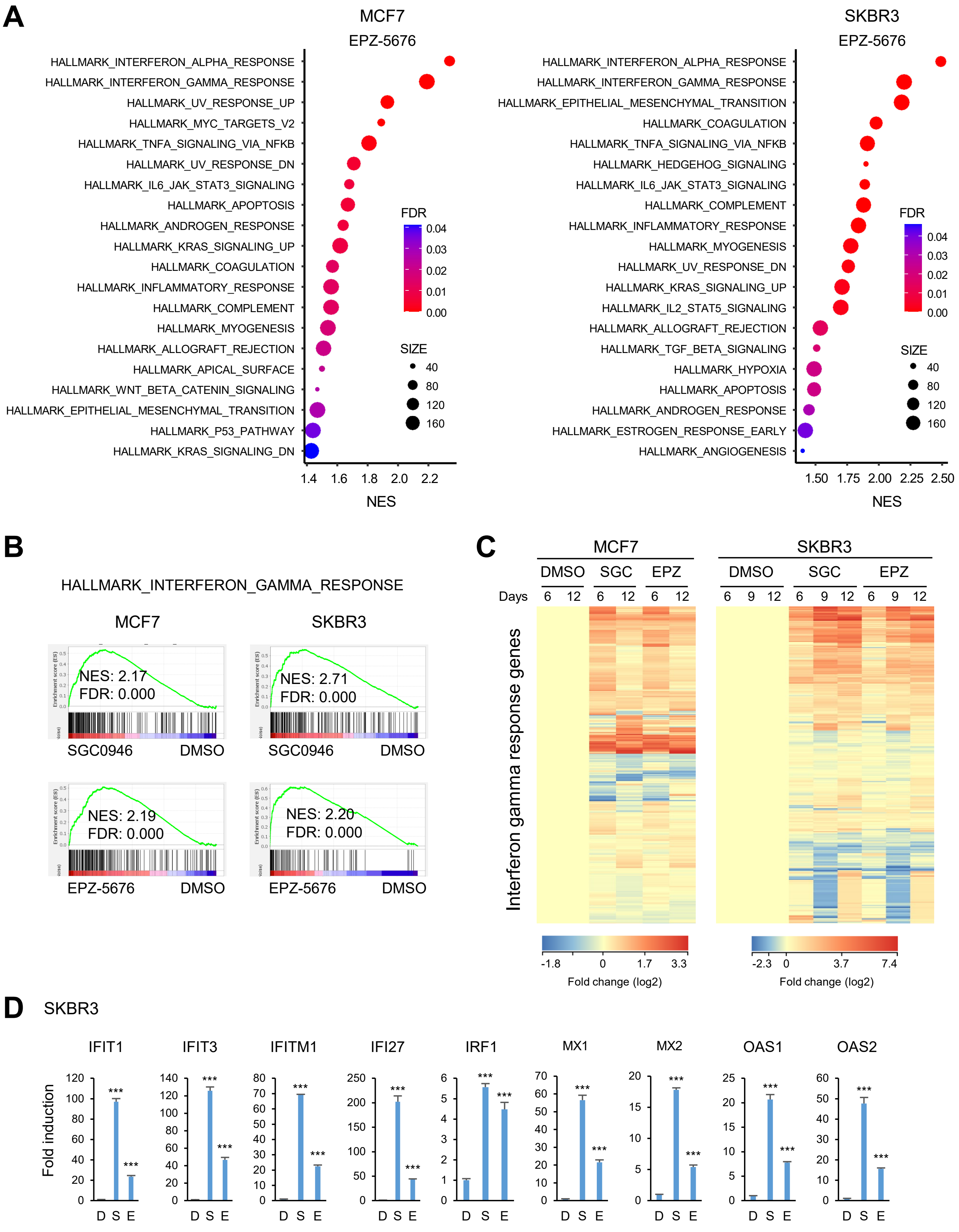

Supplementary Figure S3

DOT1L inhibition activates interferon (IFN) signaling in breast cancer cells. (A) Gene expression microarray results from MCF7 (left) and SKBR3 (right) cells treated for 6 days with DMSO or EPZ-5676. Summarized results of a GSEA of the indicated hallmark gene sets using genes upregulated by EPZ-5676 are shown. NES, normalized enrichment score; FDR, false discovery rate. (B) Results of a GSEA of the hallmark IFN-γ response gene set in breast cancer cells treated for 6 days with the indicated DOT1L inhibitor. (C) Heatmaps showing the microarray data for the hallmark IFN-γ response genes in breast cancer cells treated with DMSO, SGC0946 (SGC) or EPZ-5676 (EPZ) for the indicated periods. (D) qRT-PCR analysis of indicated IFN-related genes (IRGs) in SKBR3 cells treated with DMSO (D), SGC0946 (S) or EPZ-5676 (E) for 9 days. (n = 3). Error bars represent SDs. ***P<0.001.

Supplementary Figure S4

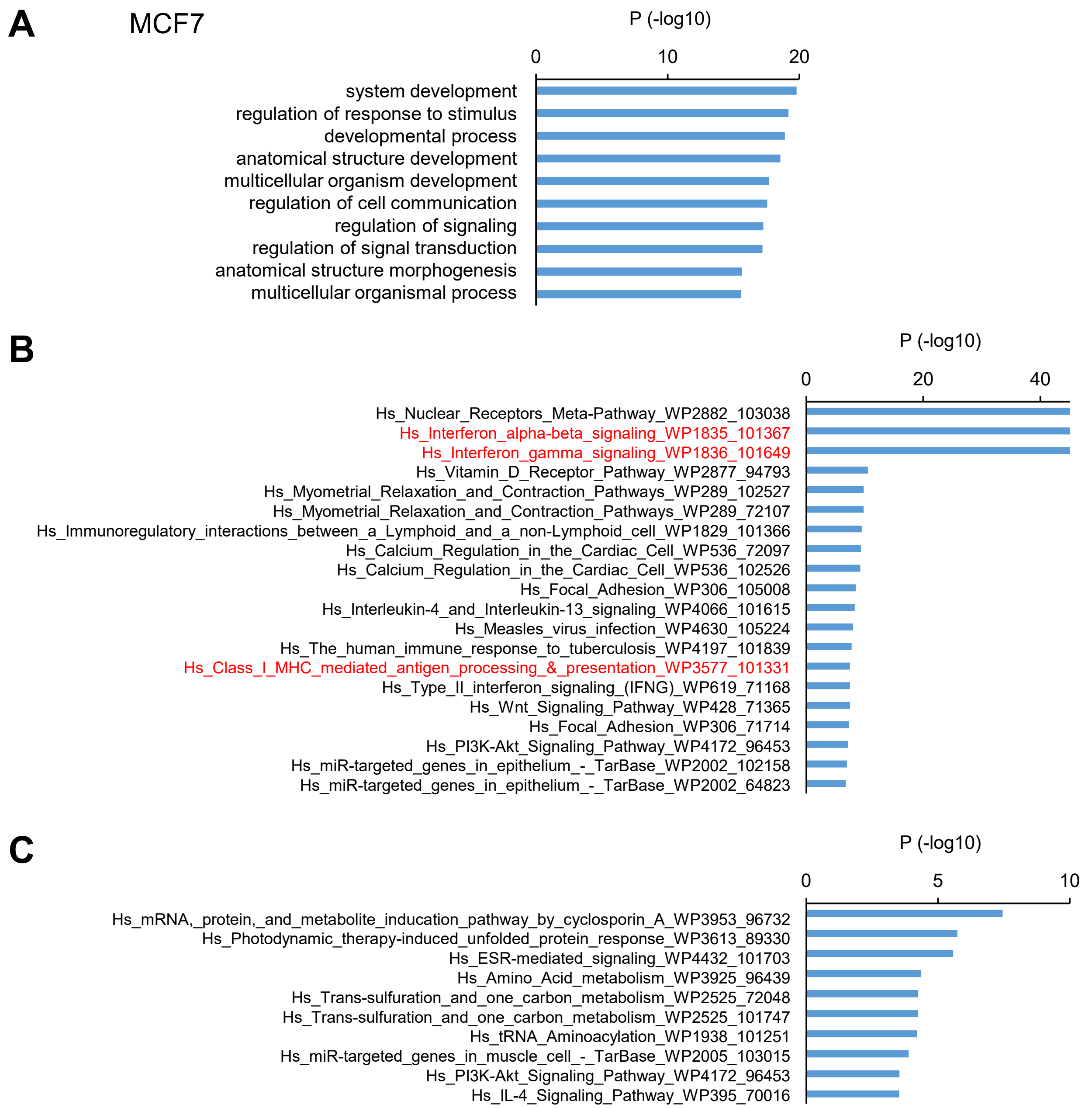

Supplementary Figure S4

Analysis of microarray data from MCF7 cells treated with DOT1L inhibitors. (A) Results of a Gene Ontology (GO) analysis using genes upregulated by DOT1L inhibition. MCF7 cells were treated for 6 days with DMSO, SGC0946 or EPZ-5676, after which gene expression microarray was performed. A series of 2310 probe sets (1924 unique genes) upregulated by the DOT1L inhibitors (>1.5-fold) were selected, after which GO analysis was performed. (B) Results of a pathway analysis using genes upregulated by DOT1L inhibition. (C) Results of a pathway analysis using genes downregulated by DOT1L inhibition. A series of 1869 probe sets (1464 unique genes) downregulated by the DOT1L inhibitors (>1.5-fold) were selected, after which pathway analysis was performed.

Supplementary Figure S5

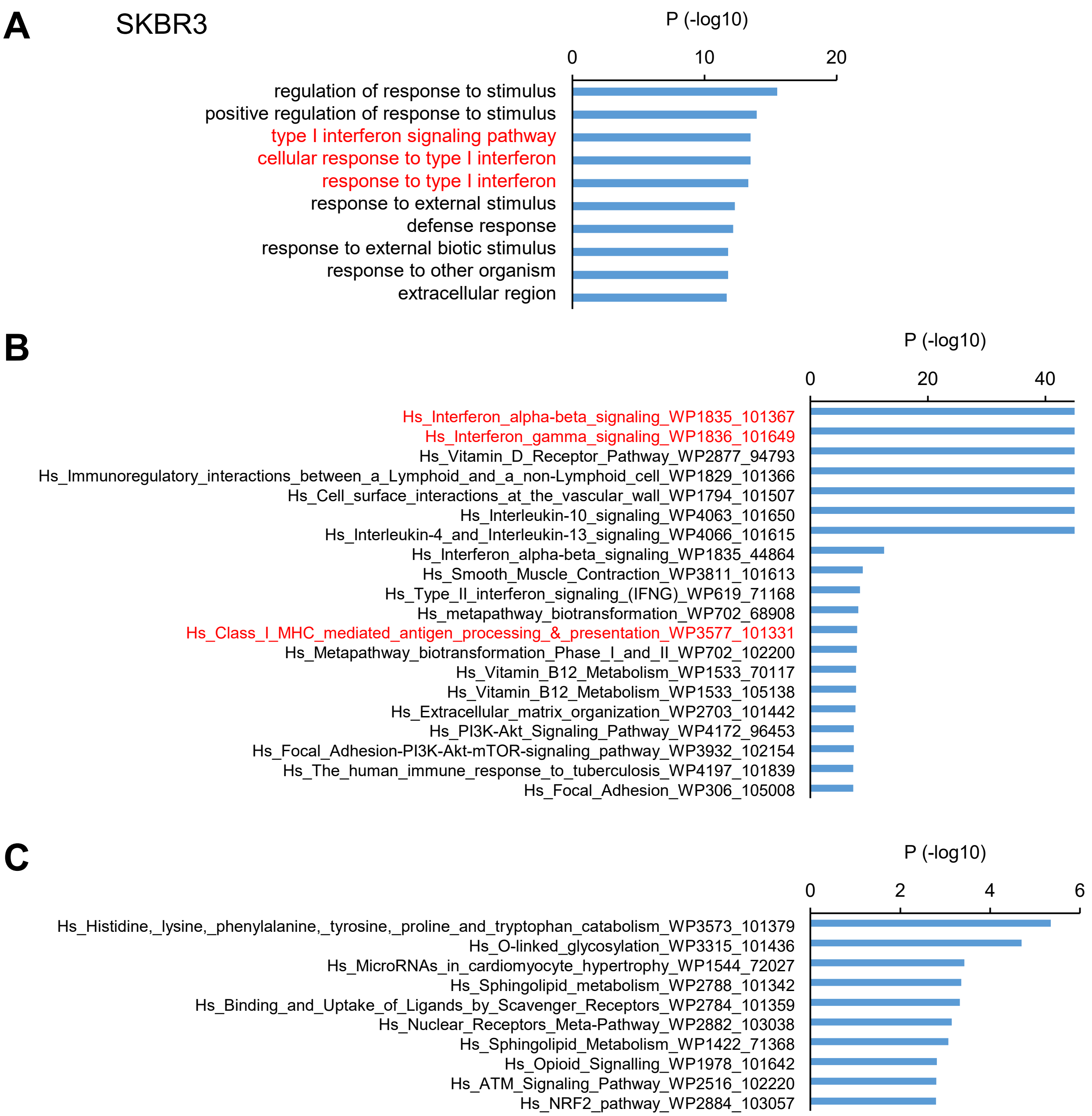

Supplementary Figure S5

Analysis of microarray data from SKBR3 cells treated with a DOT1L inhibitor. (A) Results of a Gene Ontology (GO) analysis using genes upregulated by DOT1L inhibition. SKBR3 cells were treated for 6 days with DMSO, SGC0946 or EPZ-5676, after which gene expression microarray was performed. A series of 1888 probe sets (1561 unique genes) upregulated by the DOT1L inhibitors (>1.5-fold) were selected, after which GO analysis was performed. (B) Results of a pathway analysis using genes upregulated by DOT1L inhibition. (C) Results of a pathway analysis using genes downregulated by DOT1L inhibition. A series of 1163 probe sets (930 unique genes) downregulated by the DOT1L inhibitors (>1.5-fold) were selected, after which pathway analysis was performed.

Supplementary Figure S6

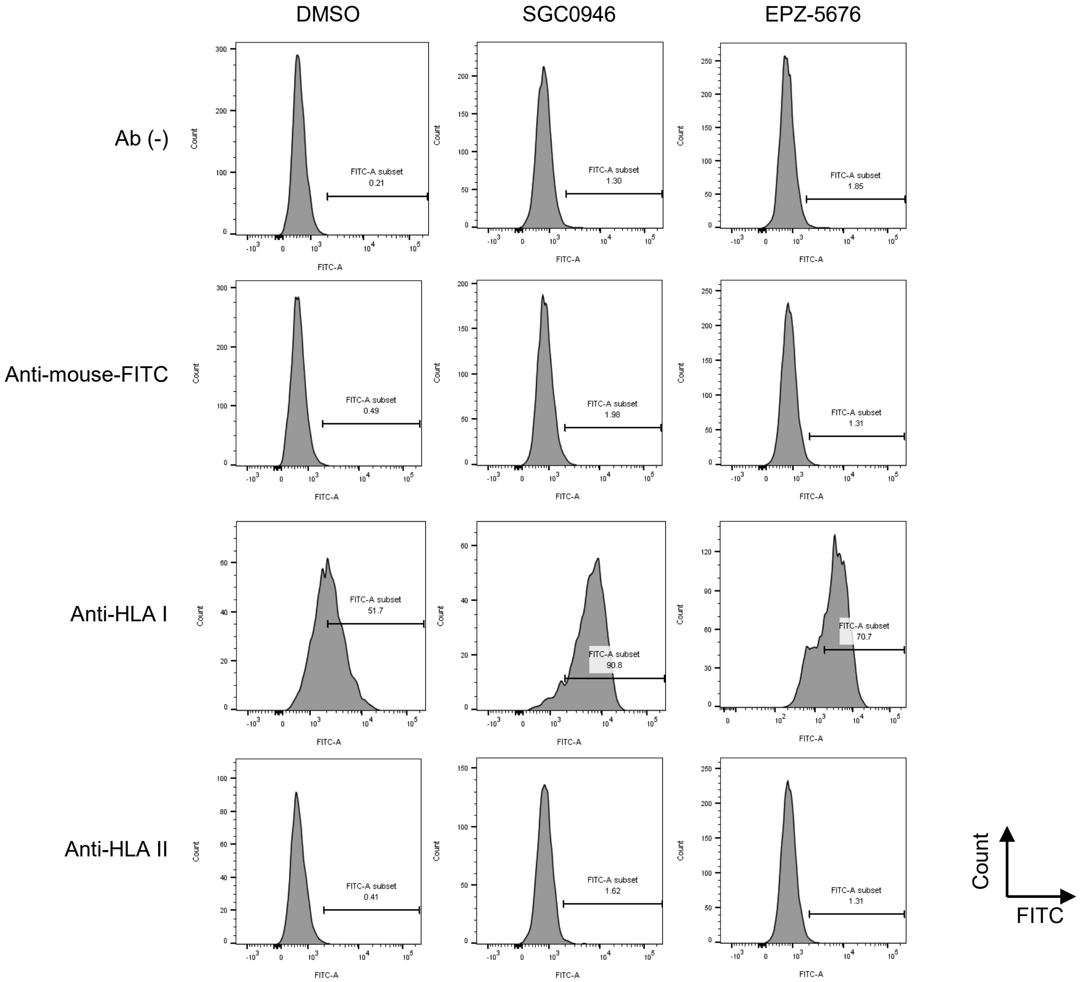

Supplementary Figure S6
DOT1L inhibition upregulates HLA expression in breast cancer cells. Shown are flow cytometry analyses of the cell surface expression of HLA class I and II molecules in MCF7 cells treated for 9 days with the indicated agents.

Supplementary Figure S7

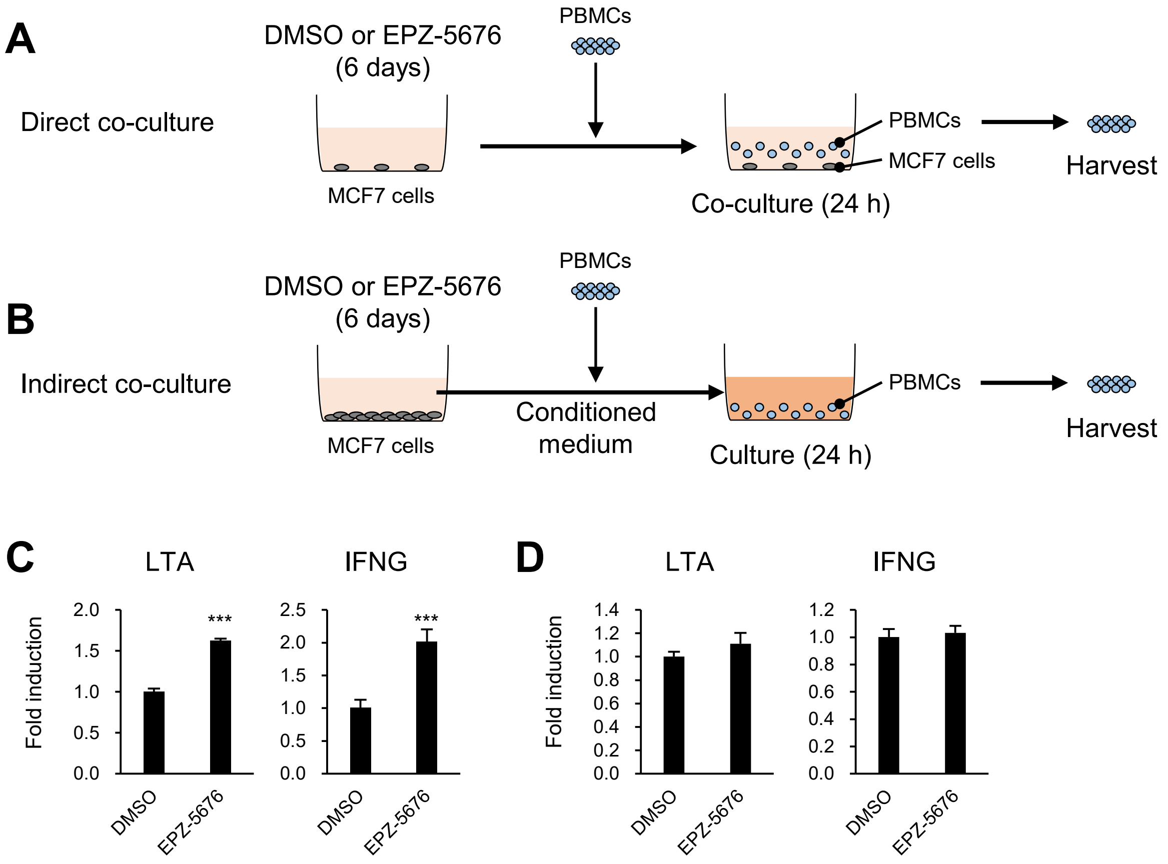

Supplementary Figure S7

DOT1L inhibitor-treated breast cancer cells may activate lymphocytes. (A) Workflow of the direct co-culture experiments. Peripheral blood mononuclear cells (PBMCs) were co-cultured with MCF7 cells that had been pre-treated with DMSO or EPZ-5676 (1 μM) for 6 days. (B) Workflow of the indirect co-culture experiments. PBMCs were cultured in medium conditioned by MCF7 cells pre-treated with DMSO or EPZ-5676 (1 μM) for 6 days. (C, D) qRT-PCR analysis of LTA and IFNG expression in PBMCs co-cultured with pre-treated MCF7 cells (C) or cultured in medium conditioned by pre-treated MCF7 cells (D). (n = 3). Error bars represent SEM. ***$P < 0.01$.

Supplementary Figure S8

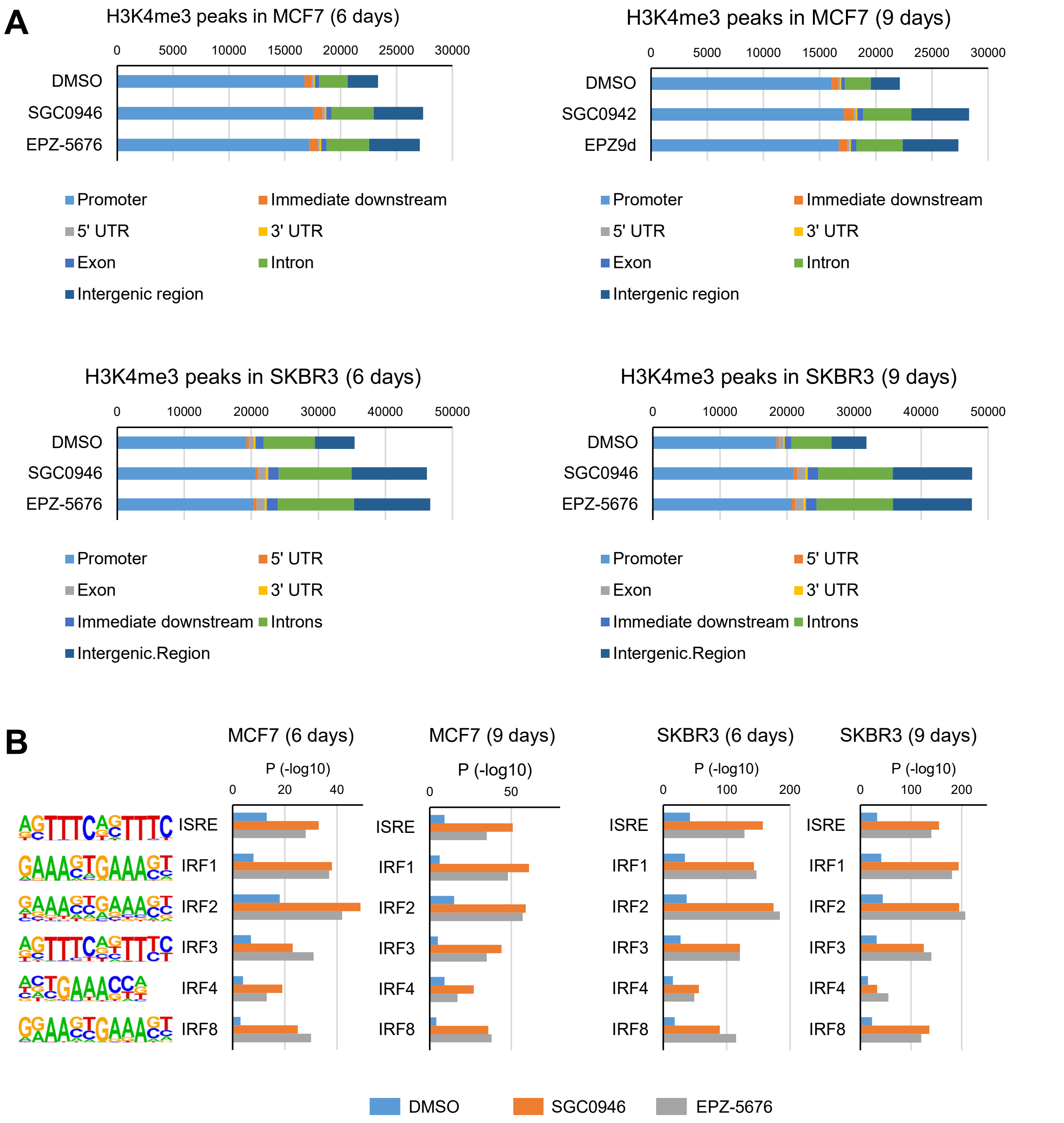

Supplementary Figure S8
ChIP-seq analysis of histone H3 lysine 4 trimethylation (H3K4me3) in breast cancer cells treated with a DOT1L inhibitor. (A) Numbers of H3K4me3 peaks and their genomic locations in MCF7 and SKBR3 cells treated for the indicated periods with DMSO, SGC0946 or EPZ-5676. (B) Motif analysis of the H3K4me3 peaks in MCF7 and SKBR3 cells treated as indicated.

Supplementary Figure S9

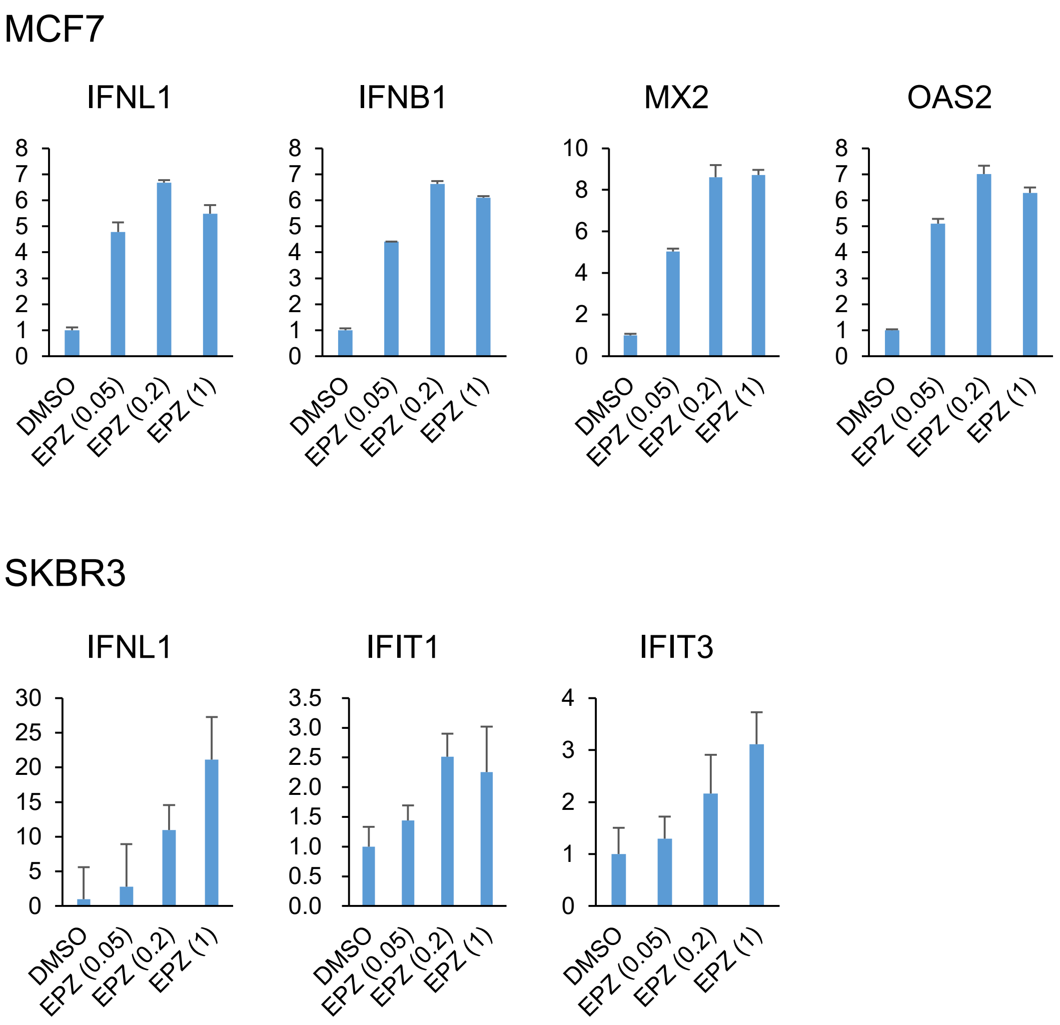

Supplementary Figure S9

Dose-dependent effects of DOT1L inhibition on the induction of IFN and IRG expression in breast cancer cells. Breast cancer cells were treated with DMSO or EPZ-5676 (0.05 μM, 0.2 μM, 1 μM) for 6 days, followed by qRT-PCR analysis of the indicated genes.

Supplementary Figure S10

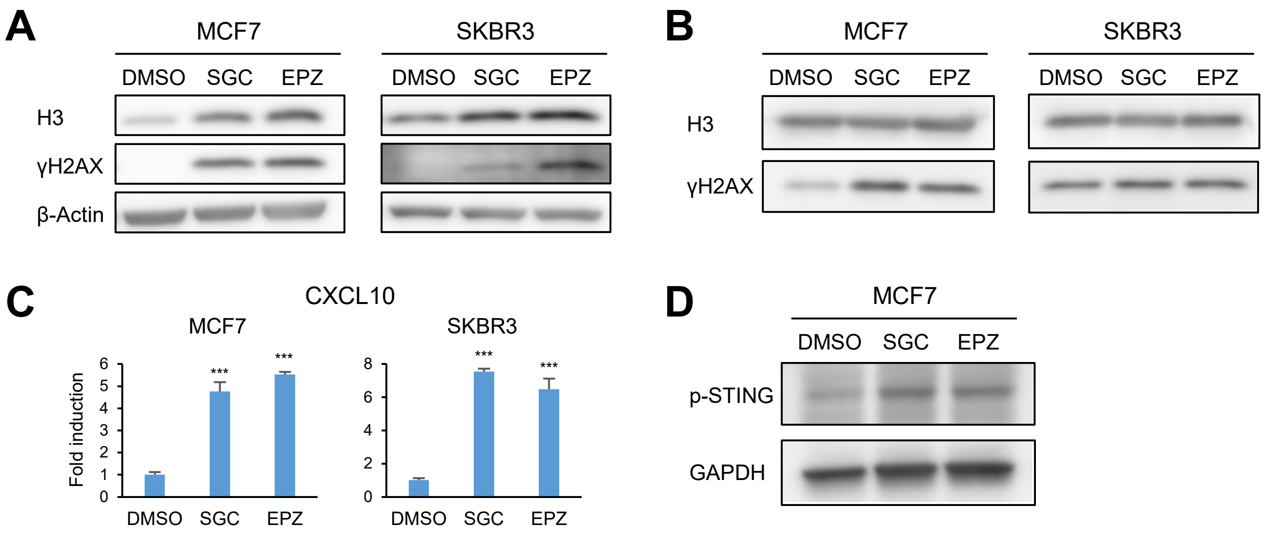

Supplementary Figure S10

DOT1L inhibition induces DNA damage in breast cancer cells. (A) Western blot analysis of histone H3 and γH2AX in the cytoplasm of MCF7 and SKBR3 cells treated for 9 days with DMSO, SGC0946 (SGC) or EPZ-5676 (EPZ). (B) Western blot analysis of histone H3 and γH2AX in the nuclear fractions of MCF7 and SKBR3 cells treated for 6 days with the indicated agents. (C) qRT-PCR of CXCL10 in breast cancer cells treated for 9 days with the indicated agents. (D) Western blot analysis of phosphorylated STING in MCF7 cells treated for 6 days with the indicated agents.

Supplementary Figure S11

A

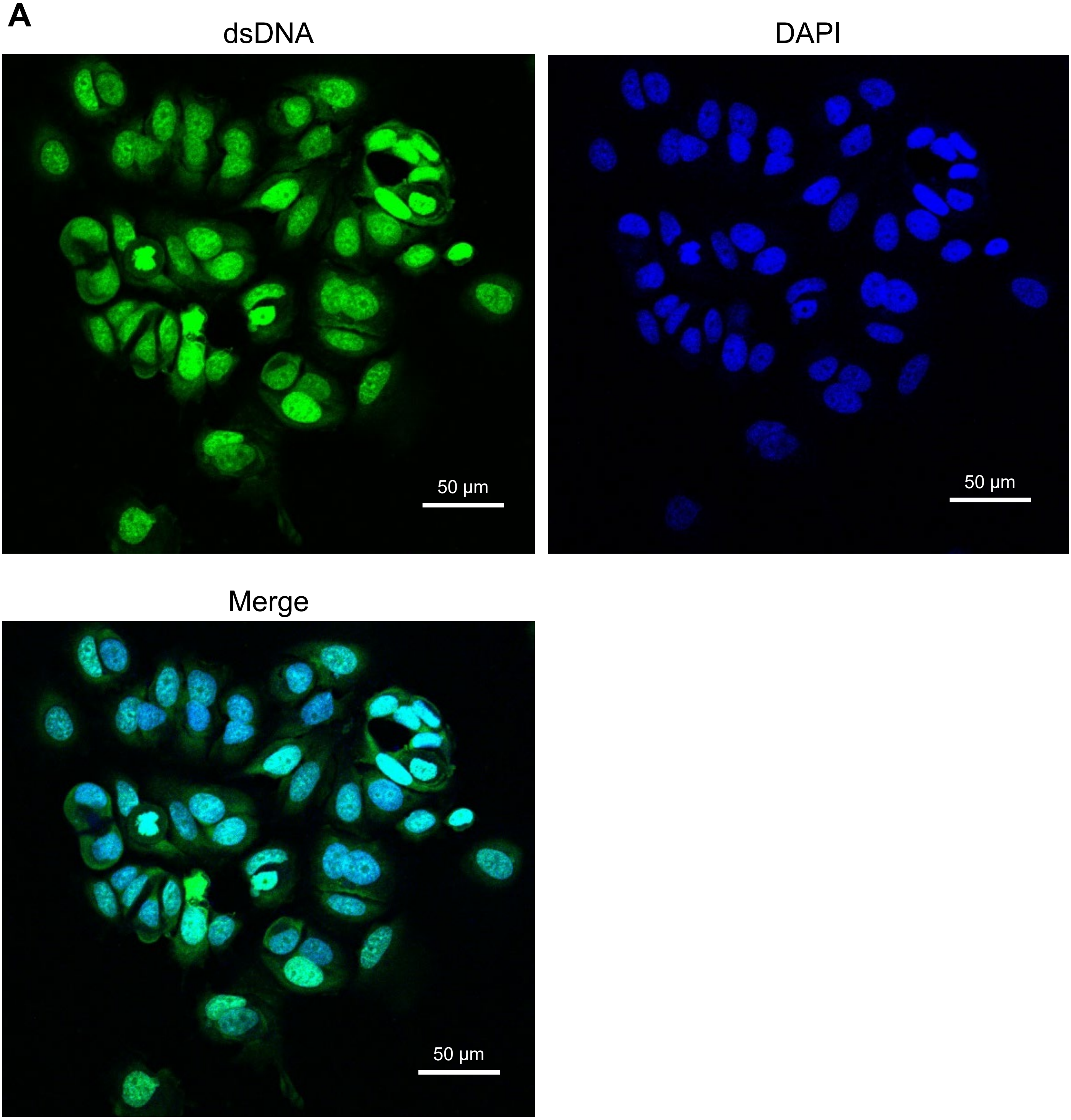

Supplementary Figure S11

B

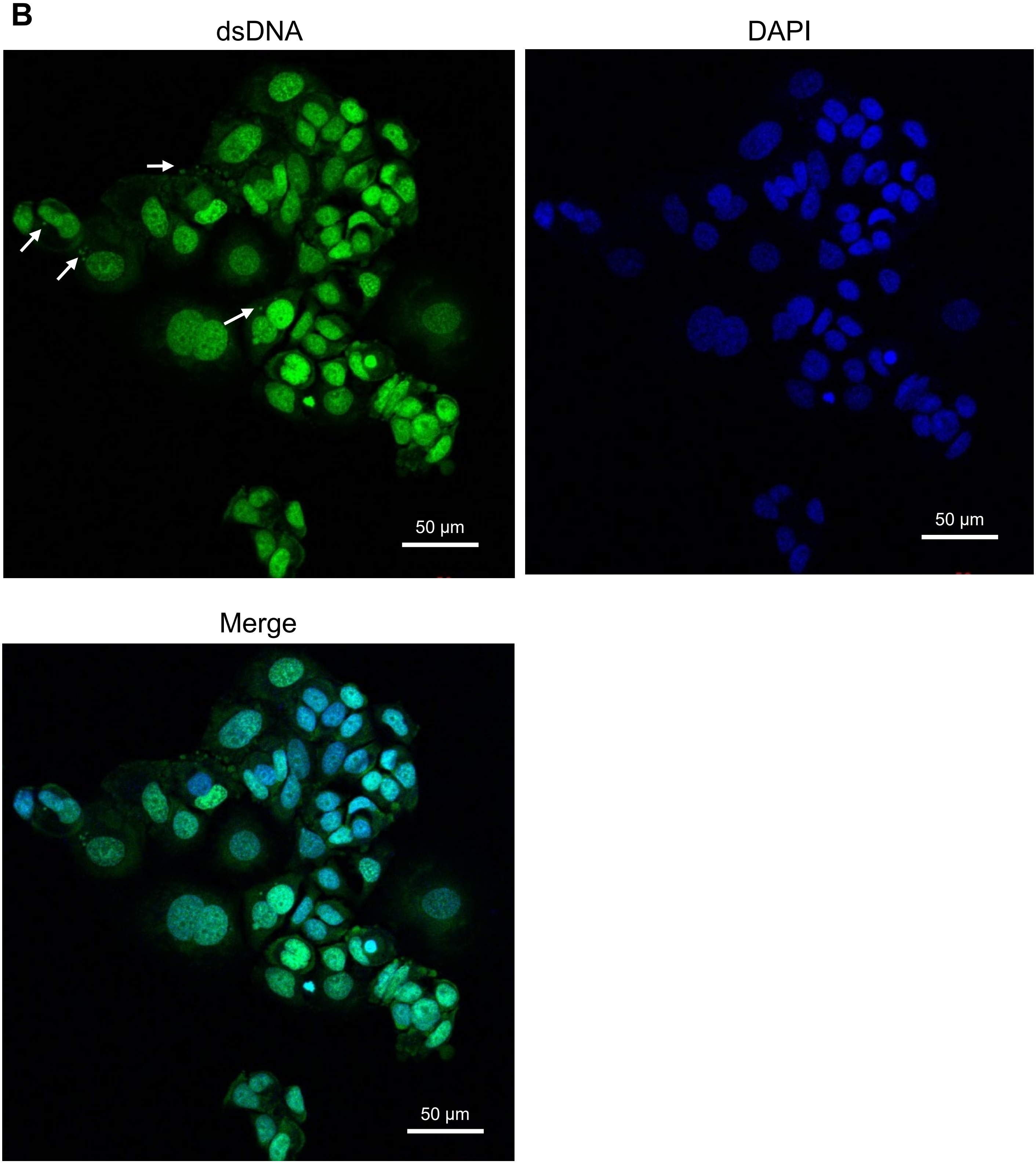

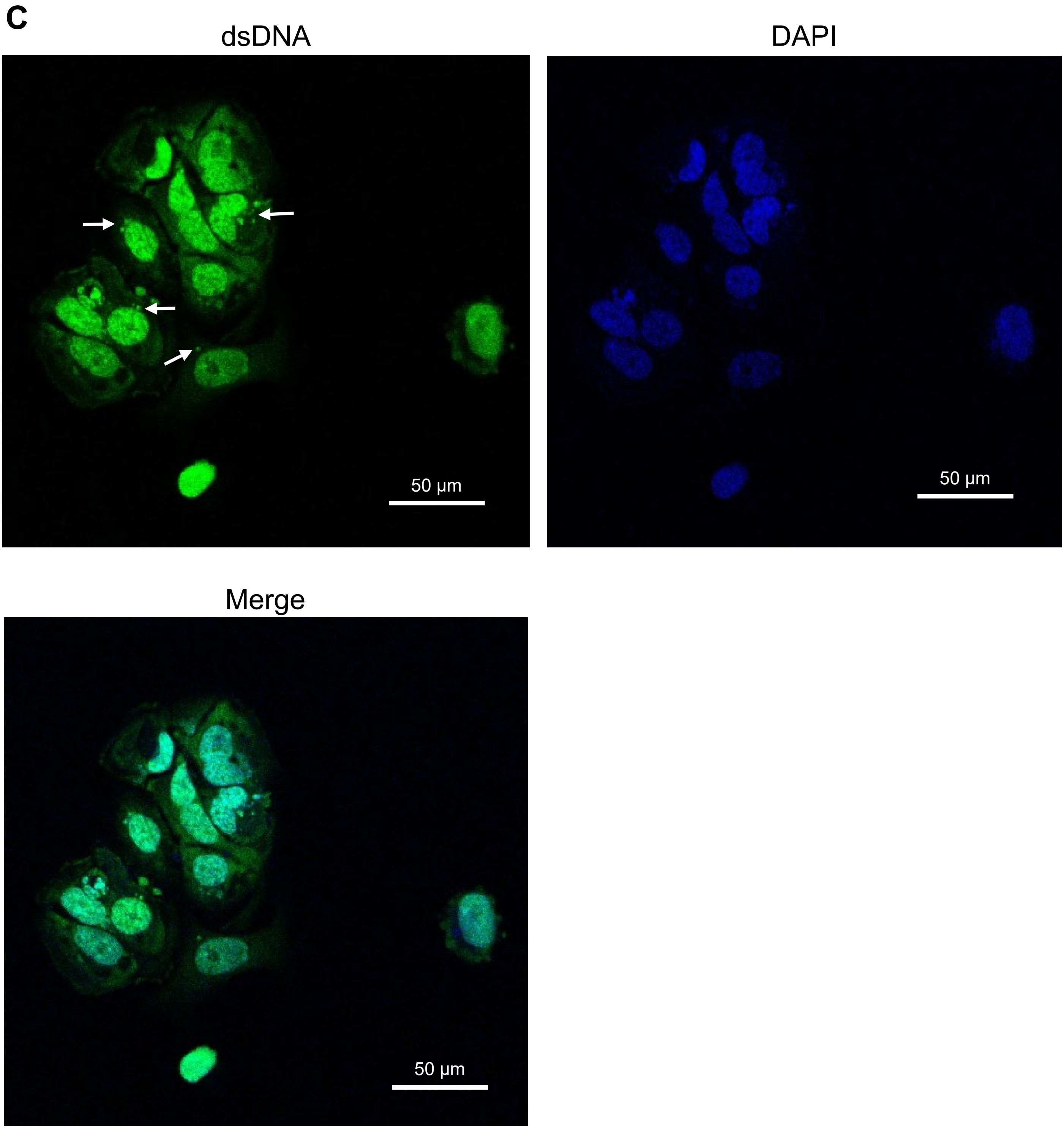

Supplementary Figure S11
Fluorescence images of double-stranded DNA (dsDNA) in MCF7 cells treated with DMSO (A), SGC0946 (B), or EPZ-5676 (C). Representative cytoplasmic DNA foci are indicated by arrows.

Supplementary Figure S12

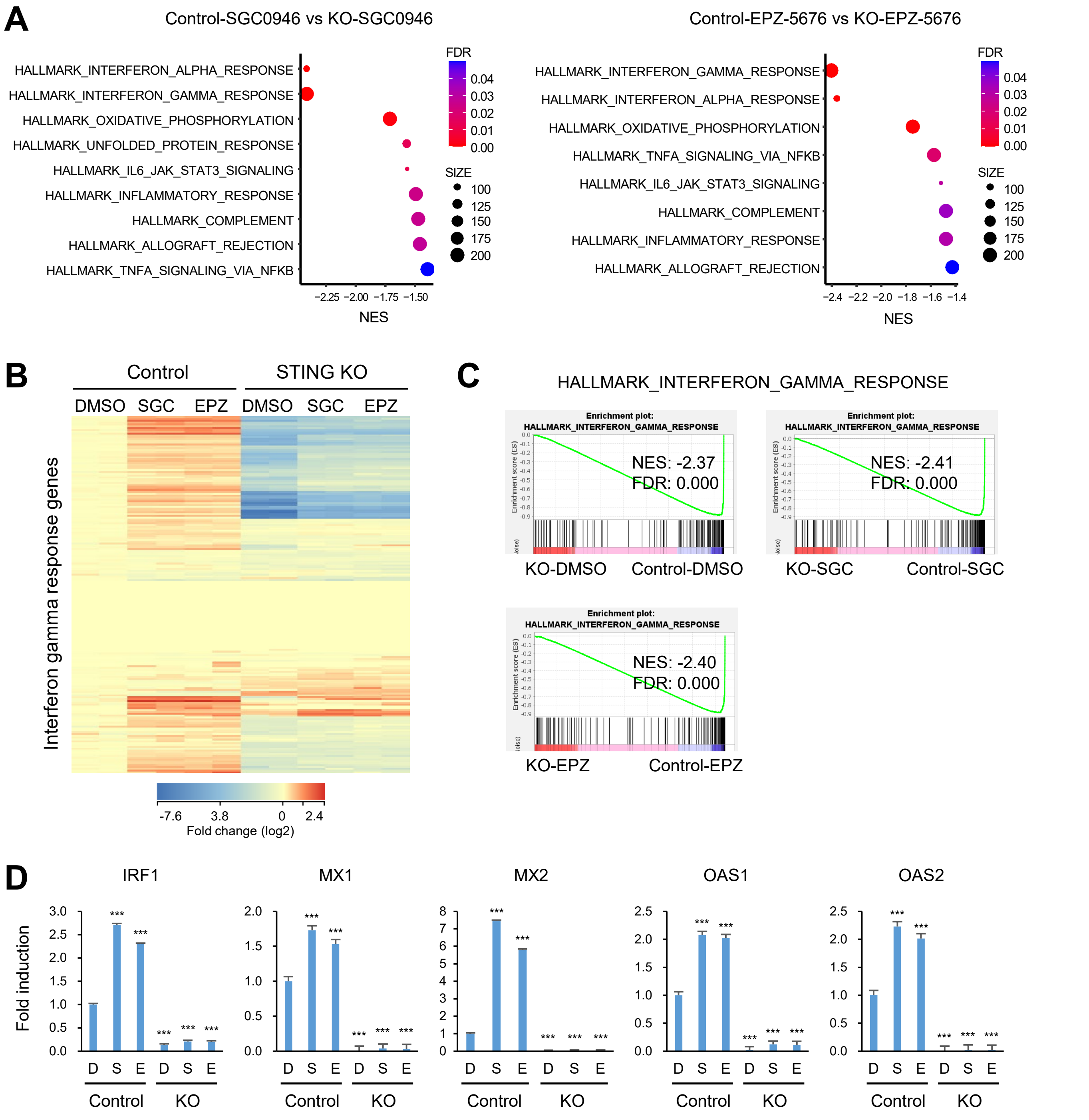

Supplementary Figure S12

The STING pathway is involved in the DOT1L inhibition-induced IFN signaling in breast cancer cells. (A) Results of RNA-seq analysis in control and STING knockout (KO) MCF7 cells treated for 6 days with SGC0946 (left) or EPZ-5676 (right). Summarized results of GSEA of indicated gene sets using genes downregulated in KO cells are shown. (B) Heatmap showing the expression levels of the hallmark IFN-γ response genes in control and STING KO MCF7 cells treated for 6 days with the indicated agents. (C) GSEA of the hallmark IFN-γ response gene set in control and STING KO MCF7 cells treated for 6 days with the indicated agents. (D) qRT-PCR analysis of the indicated IRGs in control and STING KO MCF7 cells treated for 6 days with DMSO (D), SGC0946 (S) or EPZ-5676 (E). Error bars represent SDs. ****P*<0.001.

Supplementary Figure S13

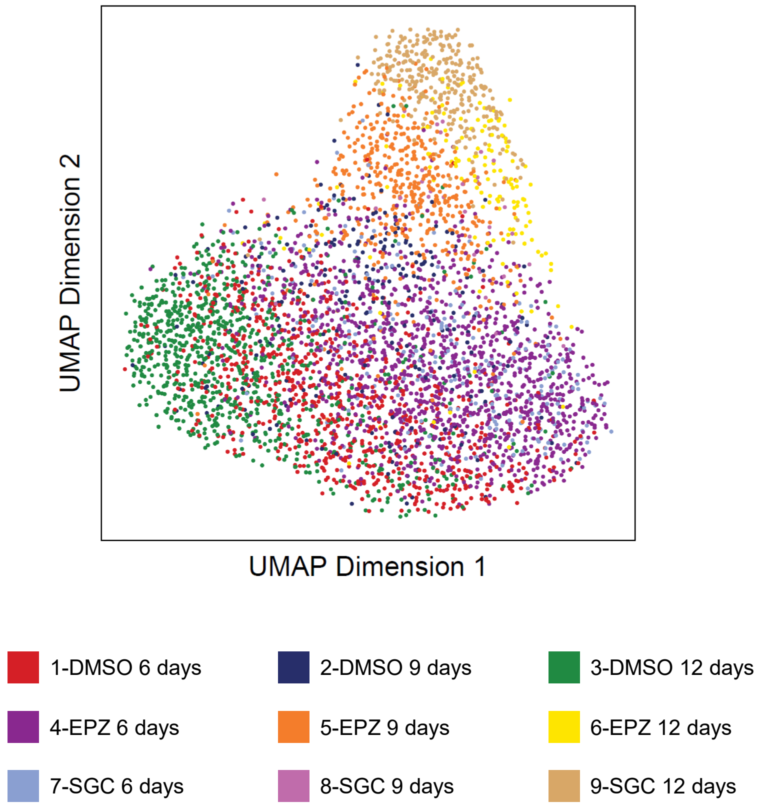

Supplementary Figure S13
Single-cell ATAC-seq (scATAC-seq) analysis in SKBR3 cells treated with a DOT1L inhibitor. Uniform manifold approximation and projection (UMAP) of the scATAC-seq data from SKBR3 cells treated for the indicated periods with DMSO, SGC0946 (SGC), or EPZ-5676 (EPZ).
