# Supplementary material for: DOT1L inhibition exerts the anti-tumor effect by activating interferon signaling in breast cancer cells
Source: Clin Epigenetics. 2025 Nov 26;17:201. doi: 10.1186/s13148-025-02017-5 (PMC12659069; doi:10.1186/s13148-025-02017-5)
Supplement: Supplementary file 2 — Supplementary Material 2 [file 13148_2025_2017_MOESM2_ESM.pdf]

Supplementary Table S1. Sequences of the primers used in this study

qRT-PCR		
IFIT1	Forward	5'-ACGGCTGCCTAATTTACAGCA-3'
	Reverse	5'-GGATAACTCCCATGTAAAGTGA-3'
IFIT3	Forward	5'-ACGGAGAAAACATCAGCTGA-3'
	Reverse	5'-TGACCTCACTCATGACTGCC-3'
IFITM1	Forward	5'-TCCACCGTGATCAACATCCA-3'
	Reverse	5'-CCAACCATCTTCCTGTCCCT-3'
IFI27	Forward	5'-GATTGCTACAGTTGTGATTGGA-3'
	Reverse	5'-CATCATCTTGGCTGCTATGGA-3'
OAS1	Forward	5'-CAAGAGCCTCATCCGCCTAG-3'
	Reverse	5'-TGTTTTCATGCTCCCTCGCT-3'
OAS2	Forward	5'-TCTATTGGATGGTCAACTACAACT-3'
	Reverse	5'-GCCATTGCCAGCATATTTTATCT-3'
MX1	Forward	5'-CAGTTACCAGGACTACGAGAT-3'
	Reverse	5'-TAGCTCATGACTGATTCCCATT-3'
MX2	Forward	5'-TCTTCGGTTTCCTCCTTTACTGA-3'
	Reverse	5'-CCATTCTCTCGGAGCATAAAATACT-3'
IFNB1	Forward	5'-CTGCAACCTTTCGAAGCCTT-3'
	Reverse	5'-AGTGGAGAAGCACAACAGGA-3'
IFNL1	Forward	5'-GGAATTGGGACCTGAGGCTT-3'
	Reverse	5'-GTGTGAAGGGGCTGGTCTAG-3'
IFNL2	Forward	5'-TAAGAGGGCCAAAGATGCCT-3'
	Reverse	5'-CTCAGCCTCCAAAGCCATG-3'
IFNG	Forward	5'-CTCTGCATCGTTTTGGGTTCTC-3'
	Reverse	5'-TCCGCTACATCTGAATGACCTG-3'
LTA	Forward	5'-AAACCTGCTGCTCACCTCATT-3'
	Reverse	5'-GGAGAGAATTGTTGCTCAAGG-3'
CXCL10	Forward	5'-GAATTTACTGAAAGCAGTTAGC-3'
	Reverse	5'-CTCTGTGTGGTCCATCCTTG-3'
ERBB2	Forward	5'-TCACCTACCTGCCCACCAAT-3'
	Reverse	5'-ACCTGCCTCACTTGGTTGTG-3'
ACTB	Forward	5'-GCCAACCGCGAGAAGATGA-3'
	Reverse	5'-AGCACAGCCTGGATAGCAAC-3'

Supplementary Table S2. Sequences of the sgRNA used in this study

Control	Forward	5’-GTTCCGCGTTACATAACTTA-3'
	Reverse	5’-TAAGTTATGTAACGCGGAAC-3'
STING1	Forward	5'-CATTACAACAACCTGCTACG-3'
	Reverse	5'-CGTAGCAGGTTGTTGTAATG-3'
