# Supplementary material for: DOT1L inhibition exerts the anti-tumor effect by activating interferon signaling in breast cancer cells
Source: Clin Epigenetics. 2025 Nov 26;17:201. doi: 10.1186/s13148-025-02017-5 (PMC12659069; doi:10.1186/s13148-025-02017-5)

Yoshido *et al*. Original images of western blots

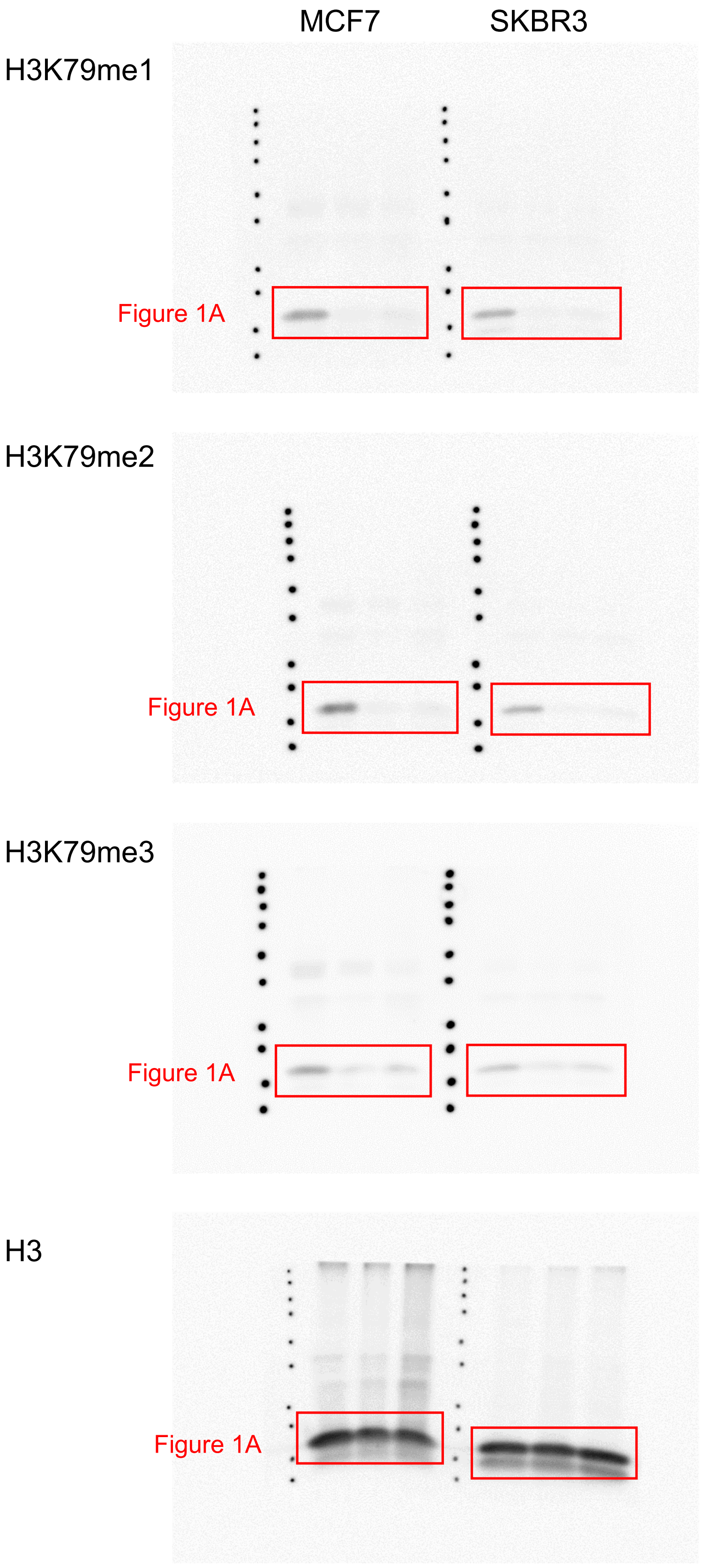

Yoshido *et al*. Original images of western blots

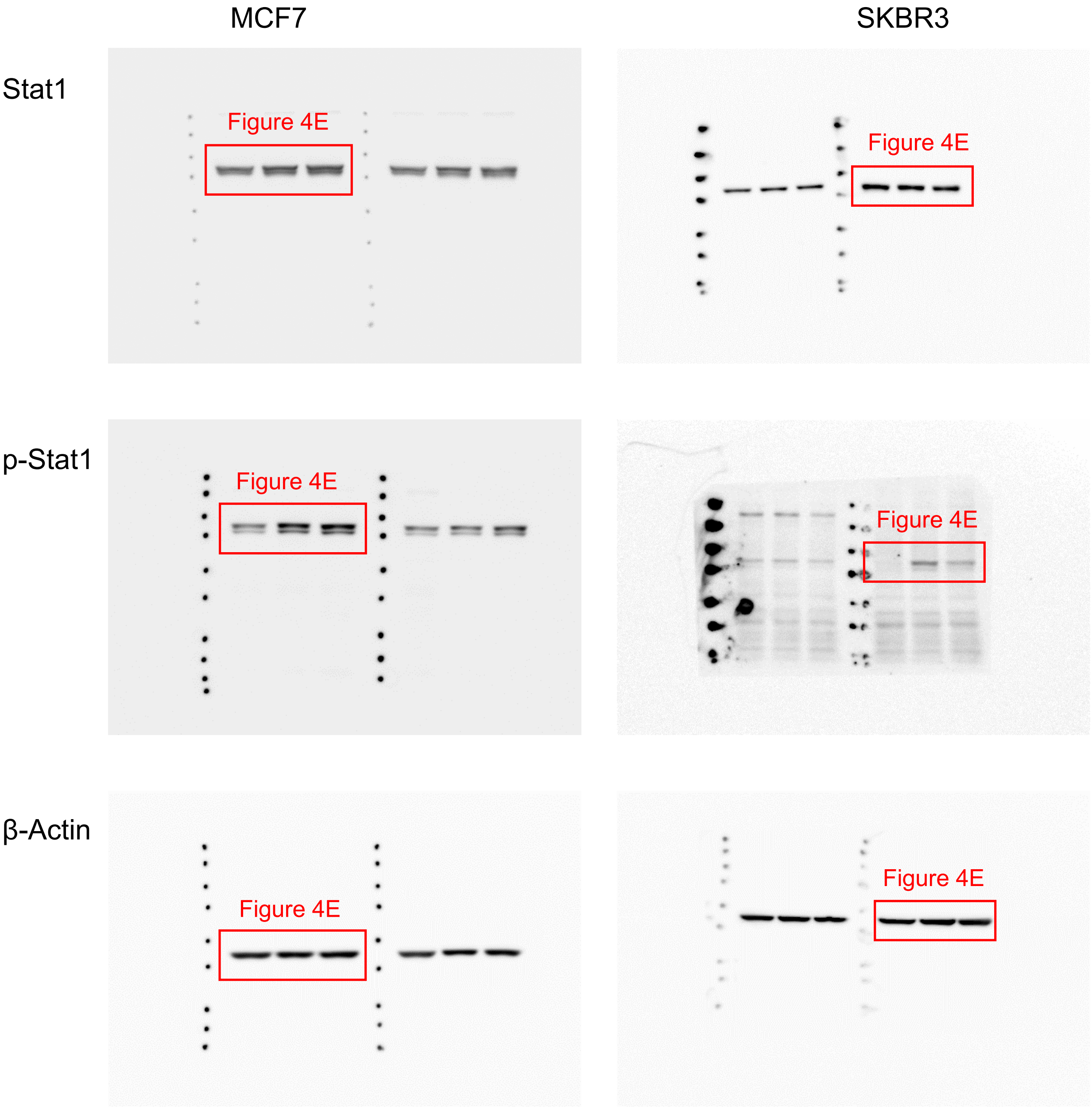

Yoshido *et al*. Original images of western blots

MCF7 SKBR3

H3

γH2AX

β-Actin

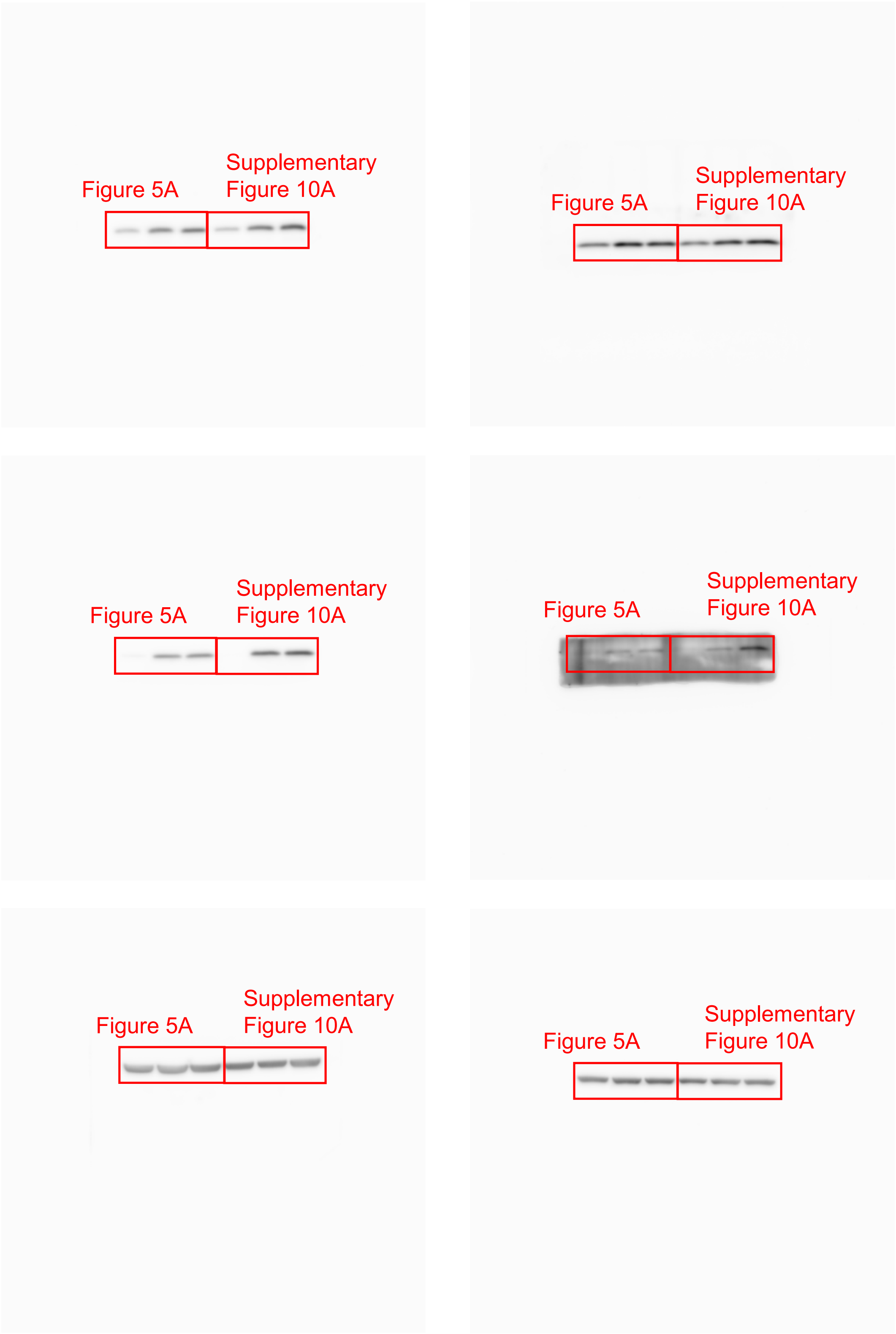

MCF7 control and STING KO cells

Stat1

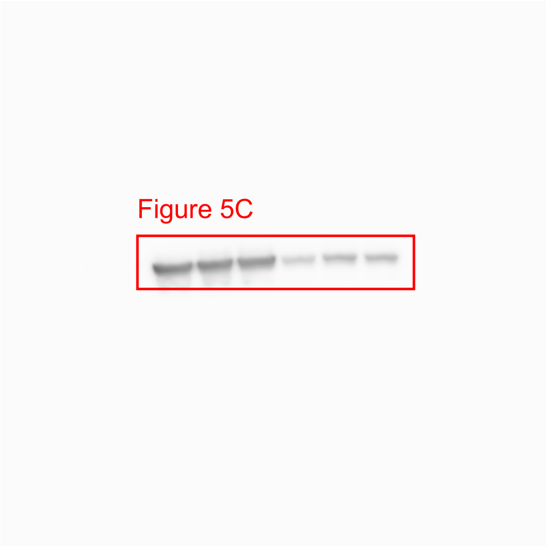

p-Stat1

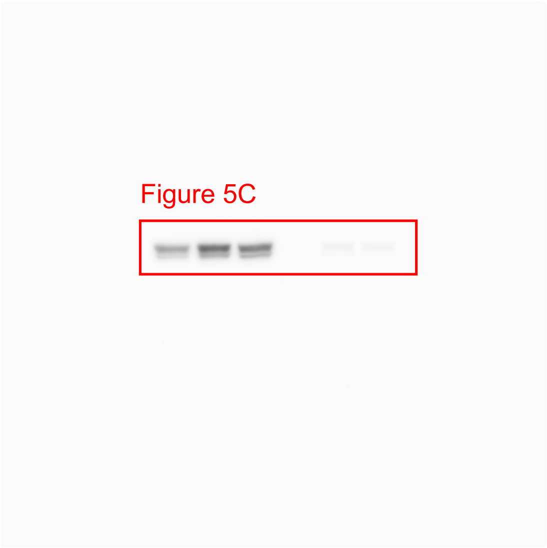

STING

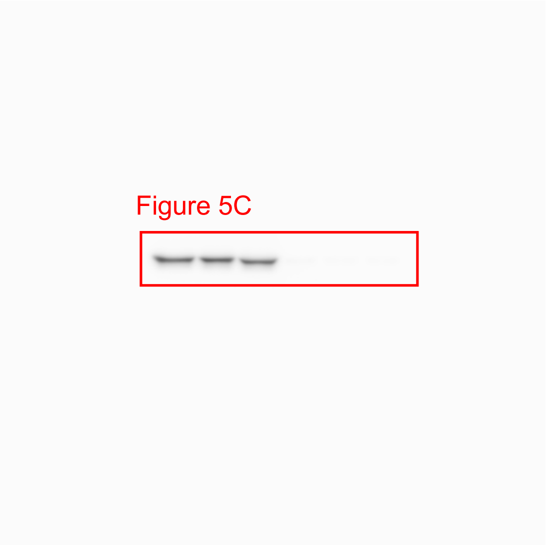

β-Actin

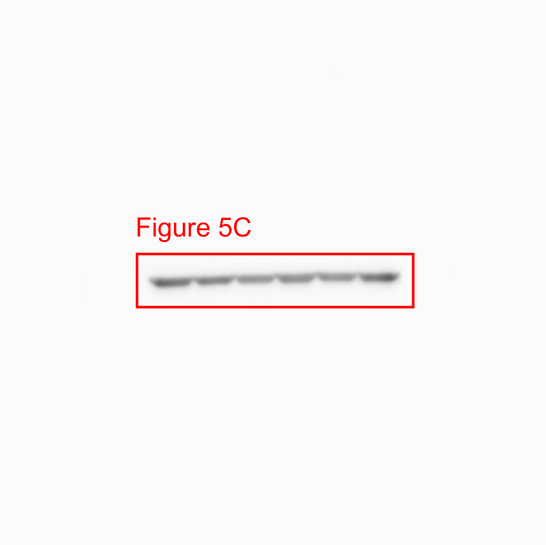

Yoshido *et al*. Original images of western blots

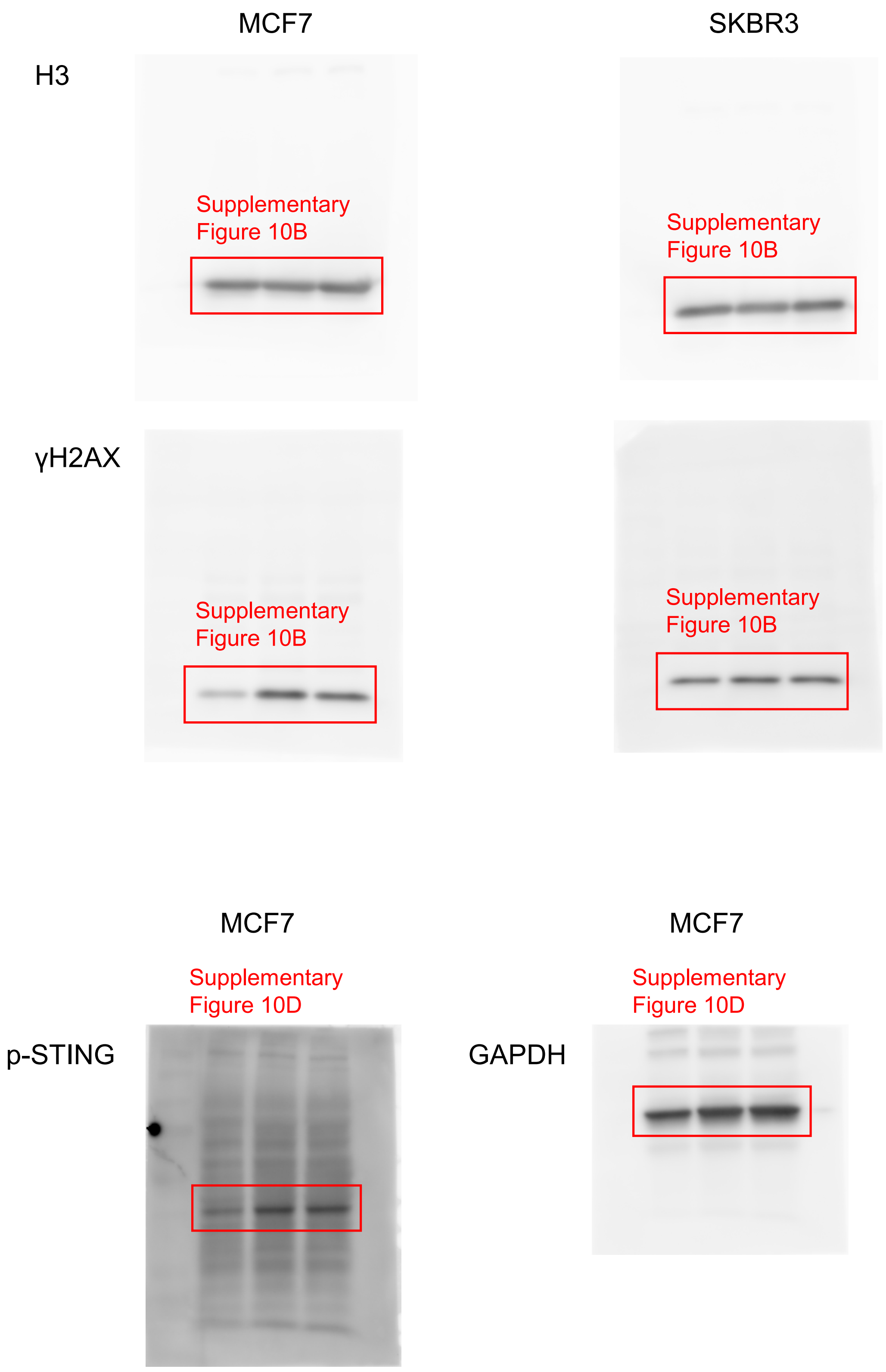

Supplement: Supplementary file 3 — Supplementary Material 3 [file 13148_2025_2017_MOESM3_ESM.pdf]
