# Supplementary material for: DOT1L inhibition exerts the anti-tumor effect by activating interferon signaling in breast cancer cells
Source: Clin Epigenetics. 2025 Nov 26;17:201. doi: 10.1186/s13148-025-02017-5 (PMC12659069; doi:10.1186/s13148-025-02017-5)
Supplement: Supplementary file 4 — Supplementary Material 4 [file 13148_2025_2017_MOESM4_ESM.docx]

**Supplementary methods**

**Isolation and culture of peripheral blood mononuclear cells**

Peripheral blood mononuclear cells (PBMCs) were isolated from a healthy donor as described previously [1]. PBMCs were cultured for 3 days in AIM V medium (Thermo Fisher Scientific) supplemented with 10% human AB serum (Biowest, Nuaillé, France) and 100 U/mL IL-2 (R&D Systems). PBMCs were then co-cultured with MCF7 cells that had been pre-treated with EPZ-5676 (1 μM) or DMSO for 6 days (PBMC:MCF7 ratio = 6:1). Alternatively, PBMCs were cultured in medium conditioned by MCF7 cells treated with EPZ-5676 (1 μM) or DMSO for 6 days. After 24 h, total RNA was extracted. Written informed consent was obtained from the donor prior to blood collection.

**Fluorescence imaging**

Breast cancer cells were treated for 6 days with 1 μM DOT1L inhibitor (SGC0946 or EPZ-5676) or DMSO. Following treatment, the cells were washed once with phosphate-buffered saline (PBS). The cells were then incubated for 2 hours in fresh medium containing Quant-iT PicoGreen dsDNA Reagent (Thermo Fisher Scientific). The cells were subsequently fixed with 4% paraformaldehyde for 5 minutes and washed again with PBS. Coverslips were mounted using Prolong Glass Antifade Mountant with NucBlue (Thermo Fisher Scientific). Images were obtained with a Zeiss LSM 780 confocal microscope system (Carl Zeiss AG, Oberkochen, Germany).

**Single-cell Assay for Transposase-Accessible Chromatin sequencing**

SKBR3 cells were treated with drugs for 6, 9 or 12 days as described above. Single-cell Assay for Transposase-Accessible Chromatin sequencing (scATAC-seq) libraries were generated using a droplet-based combinatorial indexing approach with some modifications [2]. A custom indexed-Tn5 transposase was prepared following a previously described protocol [3]. Library preparation involved tagmentation with the indexed-Tn5, followed by the use of the SureCell ATAC-Seq Library Prep Kit (Bio-Rad, Hercules, CA, USA) and the ddSEQ Single-Cell Isolator system (Bio-Rad). Q5 HiFi DNA Polymerase (New England Biolabs, Ipswich, MA, USA) and USER II Enzyme (New England Biolabs) were incorporated into the Barcode Reaction and Enzyme Reaction mixes. The finalized scATAC-seq libraries were loaded at a concentration of 1.5 pM onto a NextSeq 550 platform (Illumina), after which sequencing was conducted using the following parameters: Read 1, 118 cycles; i7 index read, 8 cycles; Read 2, 40 cycles.

The hg19 genome assembly was used for all scATAC-seq analyses. The initial processing of scATAC-seq data to generate barcoded and aligned read BAM files was performed using the ATAC‑Seq Analysis Toolkit (Bio-Rad). Sequencing data were then processed by converting the BAM files into fragment files using Rsamtools (https://bioconductor.org/packages/Rsamtools). ArchR v1.0.2 was used for further analysis unless otherwise noted [4]. Fragment files were loaded using the createArrowFiles function. Quality control analysis was conducted for each cell; cells with a transcription start site (TSS) enrichment score < 4 and number of unique fragments < 2500 were excluded. After creating arrow files for each sample using createArrowFiles, an ArchR project was generated by ArchRProject. To filter out doublets, addDoubletScores with k = 10, knnMethod = “UMAP,” LSIMethod = 1 was used. For clustering, iterative latent semantic indexing (LSI) was performed using the addIterativeLSI function with a genome-wide 500 bp tile matrix. Cells were clustered with the addClusters function, and uniform manifold approximation and projection (UMAP) embeddings were generated using the addUMAP function. Gene activity scores were visualized using the plotEmbedding function, with imputation weights calculated via the addImputeWeights function. Peak calling was conducted using the addGroupCoverage and addReproduciblePeakSet functions. Motif scores were computed using addMotifAnnotation(motifSet = "cisbp") and addDeviationMatrix. For pseudotime analysis, trajectories were inferred using the addTrajectory function, and motif enrichment along pseudotime was visualized using plotTrajectory and plotTrajectoryHeatmap.

**References**

1. Sekiguchi S, Yorozu A, Okazaki F, Niinuma T, Takasawa A, Yamamoto E, et al. ACLP Activates Cancer-Associated Fibroblasts and Inhibits CD8+ T-Cell Infiltration in Oral Squamous Cell Carcinoma. Cancers (Basel). 2023;15(17):4303.

2. Lareau CA, Duarte FM, Chew JG, Kartha VK, Burkett ZD, Kohlway AS, et al. Droplet-based combinatorial indexing for massive-scale single-cell chromatin accessibility. Nat Biotechnol. 2019;37(8):916-924.

3. Picelli S, Bjorklund AK, Reinius B, Sagasser S, Winberg G, Sandberg R. Tn5 transposase and tagmentation procedures for massively scaled sequencing projects. Genome Res. 2014;24(12):2033-2040.

4. Granja JM, Corces MR, Pierce SE, Bagdatli ST, Choudhry H, Chang HY, et al. ArchR is a scalable software package for integrative single-cell chromatin accessibility analysis. Nat Genet. 2021;53(3):403-411.
